# YY1 Silencing Induces 5-Fluorouracil-Resistance and *BCL2L15* Downregulation in Colorectal Cancer Cells: Diagnostic and Prognostic Relevance

**DOI:** 10.3390/ijms22168481

**Published:** 2021-08-06

**Authors:** Silvia Vivarelli, Luca Falzone, Saverio Candido, Benjamin Bonavida, Massimo Libra

**Affiliations:** 1 Department of Biomedical and Biotechnological Sciences, University of Catania, 95123 Catania, Italy; silvia.vivarelli7@gmail.com (S.V.); scandido@unict.it (S.C.); 2 Epidemiology and Biostatistics Unit, IRCCS Istituto Nazionale Tumori “Fondazione G. Pascale”, 80131 Naples, Italy; 3 Research Centre for Prevention, Diagnosis and Treatment of Cancer, University of Catania, 95123 Catania, Italy; 4 Department of Microbiology, Immunology and Molecular Genetics, David Geffen School of Medicine, University of California, Los Angeles, CA 90095, USA; bbonavida@mednet.ucla.edu

**Keywords:** Yin Yang 1, *BCL2L15*/Bfk, colorectal cancer, tumor-suppressor, biomarkers

## Abstract

Colorectal cancer (CRC) is characterized by genetic heterogeneity and is often diagnosed at an advanced stage. Therefore, there is a need to identify novel predictive markers. Yin Yang 1 (YY1) is a transcription factor playing a dual role in cancer. The present study aimed to investigate whether YY1 expression levels influence CRC cell response to therapy and to identify the transcriptional targets involved. The diagnostic and prognostic values of YY1 and the identified factor(s) in CRC patients were also explored. Silencing of *YY1* increased the resistance to 5-Fluorouracil-induced cytotoxicity in two out of four CRC cells with different genotypes. *BCL2L15*/Bfk pro-apoptotic factor was found selectively expressed in the responder CRC cells and downregulated upon YY1 knockdown. CRC dataset analyses corroborated a tumor-suppressive role for both YY1 and *BCL2L15* whose expressions were inversely correlated with aggressiveness. CRC single-cell sequencing dataset analyses demonstrated higher co-expression levels of both YY1 and *BCL2L15* within defined tumor cell clusters. Finally, elevated levels of YY1 and *BCL2L15* in CRC patients were associated with larger relapse-free survival. Given their observed anti-cancer role, we propose YY1 and *BCL2L15* as candidate diagnostic and prognostic CRC biomarkers.

## 1. Introduction

Colorectal Cancer (CRC) represents about 10% of the overall diagnosed tumors [1]. Although within the last ten years the survival rate has increased thanks to an augmented adhesion to screening and preventive measures, the mortality still remains significant [2]. Given the high intrinsic genomic instability, CRC shows an elevated inter-patient and intra-tumor heterogeneity, both associated with poorer outcomes [3]. In order to interconnect several existing gene expression-based CRC classifications, in 2015, an international consortium was formed and, upon extended analysis, such classifications were merged to categorize CRC into four distinguished consensus molecular subtypes (CMS): CMS1 (microsatellite instability, immune), CMS2 (canonical), CMS3 (metabolic), CMS4 (mesenchymal) [4]. Currently, CMS grouping is the most robust system to correlate distinct molecular features with clinical stratification. Nevertheless, a constant refinement of the CMS classification through the discovery of novel molecular features may lead to an increase in their predictive impact [5]. These molecular features largely influence tumor aggressiveness, metastasis formation, development of drug resistance, as well as relapses over the years [6]. To ameliorate outcomes, it is important to characterize the molecular mechanisms orchestrating CRC pathogenesis, which, in turn, will allow the improvement of diagnostic, prognostic and therapeutic strategies in this era of personalized medicine [7].

Yin Yang 1 (YY1) is a conserved C_2_H_2_-type Zinc finger transcription factor regulating the expression of about 7% of the human genes, thus affecting a number of cellular functions, including survival [8,9]. YY1 can modulate the transcription of target genes directly by binding their regulatory regions [9]. Alternatively, the transcriptional activity of *YY1* might rely on indirect interactions, either with transcriptional co-activators/co-repressors or with chromatin modulating enzymes [10]. In oncology, YY1 plays a controversial role [10]. Regarding CRC, the majority of reports indicate that YY1 might promote tumorigenesis. Additionally, YY1 could be anti-tumorigenic, either alone or in association with other co-factors [11,12,13,14,15,16,17,18,19,20,21]. For instance, YY1 in association with INO80 could bind the promoter of the *BCCIP* tumor suppressor gene and promote its expression in CRC cells [20,21,22]. Moreover, a specific transactivating domain of YY1 was found able to induce P21 pro-apoptotic gene expression in CRC cellular models [13].

The aims of this study were: (1) To define how YY1 may influence CRC cellular response to anti-cancer treatment and to further identify potential transcriptional targets of YY1 and (2) To evaluate in CRC patients the diagnostic and prognostic potential of the *YY1* gene and/or identified transcriptional target(s). In vitro studies suggested a tumor-suppressive role for both YY1 and the hereby identified pro-apoptotic factor *BCL2L15*/Bfk, in two out of four CRC cell lines with different mutational genotypes. These findings were further validated by bioinformatics analyses of CRC patients’ datasets which suggested that both *YY1* and *BCL2L15* might represent new diagnostic biomarkers of prognostic significance.

## 2. Results

### 2.1. YY1 Is Heterogeneously Expressed in CRC Cancer Cells and Its Silencing Does Not Affect Cellular Growth and Viability

**Figure 1:**
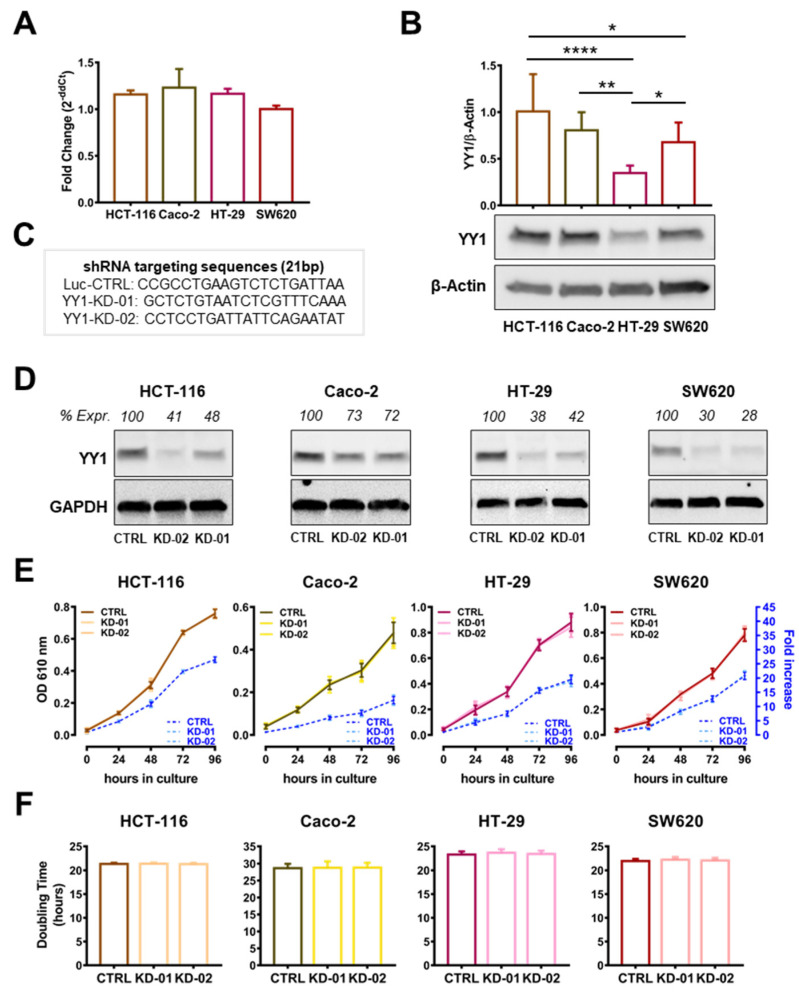
*YY1* silencing does not affect CRC cellular growth and viability. (**A**) q-RT-PCR analysis of *YY1* expression in the four CRC cell lines (GAPDH used as housekeeping); data expressed as 2^−ddCt^ and compared with SW620 normalized expression. (**B**) Immunoblot of YY1 (60 KDa) and β-Actin (42 KDa) proteins expression in the four CRC cell lines, densitometry analysis of YY1 expression (normalized to β-Actin). (**C**) shRNA targeting sequences against luciferase (CTRL) and *YY1* (KD-01 and KD-02) transcripts. (**D**) Immunoblot of YY1 and GAPDH (37 KDa) proteins expression in HCT-116, Caco-2, HT-29 and SW620 clones (CTRL, KD-02, KD-01), densitometry analysis of YY1 expression (as percentage compared to CTRL). (**E**) MTT-based growth curve of the CTRL, KD-01 and KD-02 HCT-116, Caco-2, HT-29 and SW620 CRC cells (left y-axis: OD at 610 nm over days in culture; right y-axis: fold increase in the OD over the baseline). (**F**) Doubling time of the CTRL, KD-01 and KD-02 HCT-116, Caco-2, HT-29 and SW620 CRC cells. Values are presented as Mean  ±  SD. * *p* < 0.05; ** *p*  <  0.01; **** *p*  <  0.0001; no asterisk = not significant.

Four human CRC cell lines, HT-29, SW620, HCT-116 and Caco-2, each with a different mutational background, were selected for the study in an effort to delineate the correlation between the genetic background and the expression of *YY1* (Supplementary Table S1). To measure *YY1* expression at the transcriptional level, a q-RT-PCR method was performed and, as shown in Figure 1A, the relative expression level of YY1 was comparable in all cells. Whereas, the densitometry analysis in Figure 1B showed that YY1 protein expression is heterogeneous and the HT-29 cells expressed the lowest level of YY1.

To test the function played by YY1 in the selected CRC cell lines, three sh-RNA-stably transduced clones were selected: KD-01 and KD-02, expressing shRNA targeting *YY1*, and CTRL, carrying a non-targeting shRNA (Figure 1C). To measure the efficiency of silencing, YY1 protein expression was analyzed in CTRL, KD-01 and KD-02 clones. As shown by the immunoblot and relative densitometry analyses reported in Figure 1D, for each of the CRC cell lines, KD-01 and KD-02 cells showed significantly reduced/absent YY1 expression, compared to their unsilenced CTRL, although with a different silencing efficiency, which is cell line-dependent, being the Caco-2 cells the less efficiently silenced.

To evaluate whether *YY1* silencing affects CRC cellular growth, MTT growth experiments were performed. The growth curves in Figure 1E overlapped for CTRL, KD-01 and KD-02 for all four cell lines tested. Likewise, the calculated doubling times showed no significant differences between the YY1-KD and CTRL cells (Figure 1F). In summary, the results in Figure 1 demonstrated that *YY1* silencing did not affect the cellular growth of the HCT-116, Caco-2, HT-29 and SW620 CRC cell lines.

### 2.2. YY1 Silencing Protects HT-29 and SW620 CRC Cells against 5-FU-Induced Cytotoxicity

**Figure 2:**
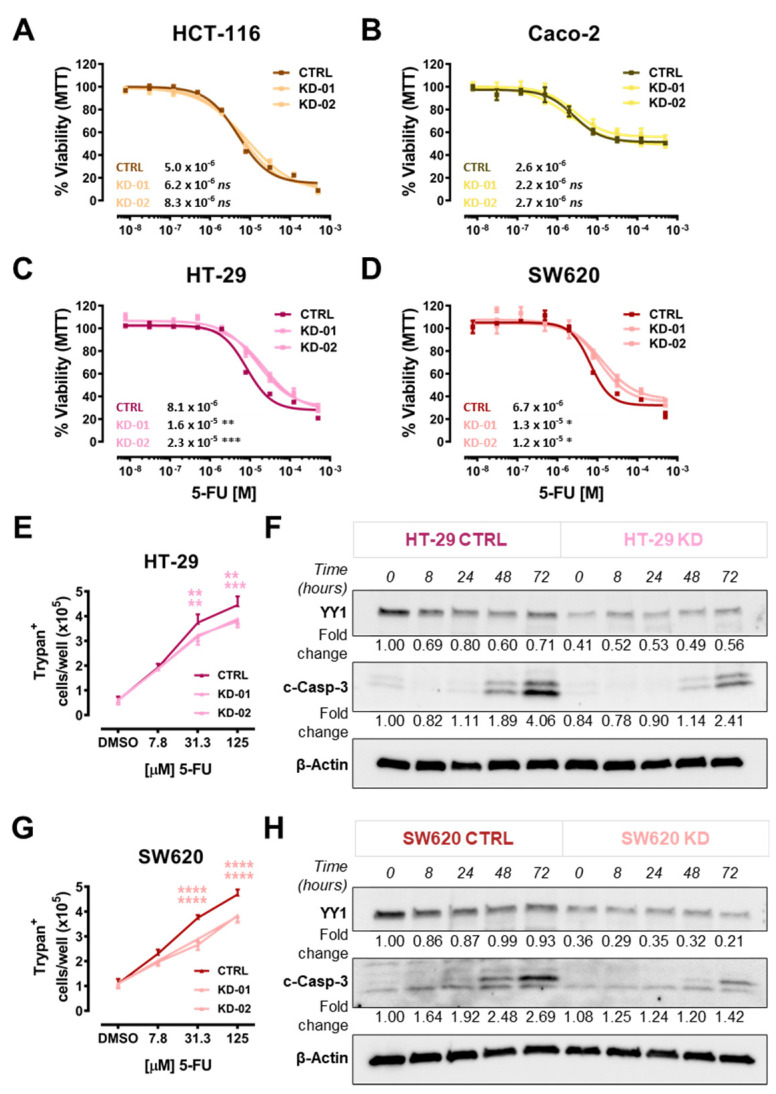
*YY1* silencing increases viability and reduces apoptosis in HT-29 and SW620 CRC cells. 5-FU concentration-response curves of: (**A**) HCT-116 (CTRL, KD-01, KD-02). (**B**) Caco-2 (CTRL, KD-01, KD-02). (**C**) HT-29 (CTRL, KD-01, KD-02). (**D**) SW620 (CTRL, KD-01, KD-02). (**E**) Trypan blue count of HT-29 (CTRL, KD-01, KD-02) at 0 (DMSO only mock control), 7.8, 31.3, 125 µM, 5-FU. (**F**) Immunoblot and densitometry of HT-29 (CTRL, KD-01) treated with 30 µM 5-FU, from 0 to 72 h. Signal detected for YY1 (60 KDa), cleaved Caspase 3 (c-Casp-3; two bands at 17 and 19 KDa) and β-Actin (normalization control, 42 KDa). (**G**) Trypan blue count of SW620 (CTRL, KD-01, KD-02) at 0 (DMSO only mock control), 7.8, 31.3, 125 µM, 5-FU. (**H**) Immunoblot and densitometry of SW620 (CTRL, KD-01) treated with 30µM 5-FU, from 0 to 72 h. Signal detected for *YY1*, c-Casp-3 and β-Actin. Values are presented as Mean  ±  SD. * *p* < 0.05; ** *p* < 0.01; *** *p* < 0.001; **** *p <* 0.0001.

To assess whether *YY1* silencing might affect the response to a cytotoxic insult, the CRC cell lines were treated with the antimetabolite 5-FU. In particular, concentration-response experiments were set up for the CTRL, KD-01 and KD-02 cells, generated from HCT-116, Caco-2, HT-29 and SW620 CRC lines, using the MTT assay as viability metabolic readout. As a result, the HCT-116 and Caco-2 cell lines did not show any difference between the KD and CTRL (Figure 2A,B). Additionally, Caco-2 was highly resistant to 5-FU-induced cytotoxicity (maximal viability reduction about 50%, Figure 2B). In contrast, the HT-29 and SW620 cell lines, when silenced for *YY1*, were significantly less sensitive to 5-FU compared with their unsilenced controls. Accordingly, for both HT-29 and SW620, *YY1* KD-01 and KD-02 clones showed a significant 10-times higher IC_50_ than their CTRL cells (Figure 2C,D).

To further assess whether the detected increase in viability of *YY1* silenced clones was linked with a decrease in cell death, a concentration-response titration with trypan blue dye exclusion assay was performed in HT-29 and SW620 responders. It was found that the number of dead cells per well increased as a function of augmented 5-FU concentrations for both HT-29 (Figure 2E) and SW620 (Figure 2G).

To further evaluate whether the reduced cell viability correlated with a reduction in apoptosis, a kinetic time-course experiment was performed. HT-29 and SW620 cells were treated with 30 µM 5-FU, and protein samples were harvested at several time points (from 0 to 72 h) and analyzed by immunoblots. Cleaved caspase 3 (c-Casp-3) protein detection was used as an apoptosis readout. The detected c-Casp-3 signal was greater in cells that retain YY1 expression (CTRL) compared with the *YY1*-KD, as shown by the densitometry values (Figure 2F,H). Overall, both HT-29 and SW620, when silenced for *YY1*, significantly halved the apoptotic response upon 72 h of 5-FU treatment (Figure 2F,H).

In summary, the above findings demonstrated that YY1 regulated the sensitivity of both HT-29 and SW620 to 5-FU cytotoxicity, as both the CRC cell lines, when silenced for YY1, were significantly less sensitive to 5-FU compared to non-silenced CTRLs. The differential response was observable only for HT-29 and SW620 cells, but not for HCT-116 and Caco-2 cells. This result suggested that YY1 might act as a tumor suppressor selectively in the two responder CRC cell lines.

### 2.3. YY1 Silencing Is Associated with the Downregulation of the Tumor-Suppressor BCL2L15 (Bfk) in HT-29 and SW620 CRC Cells

**Figure 3:**
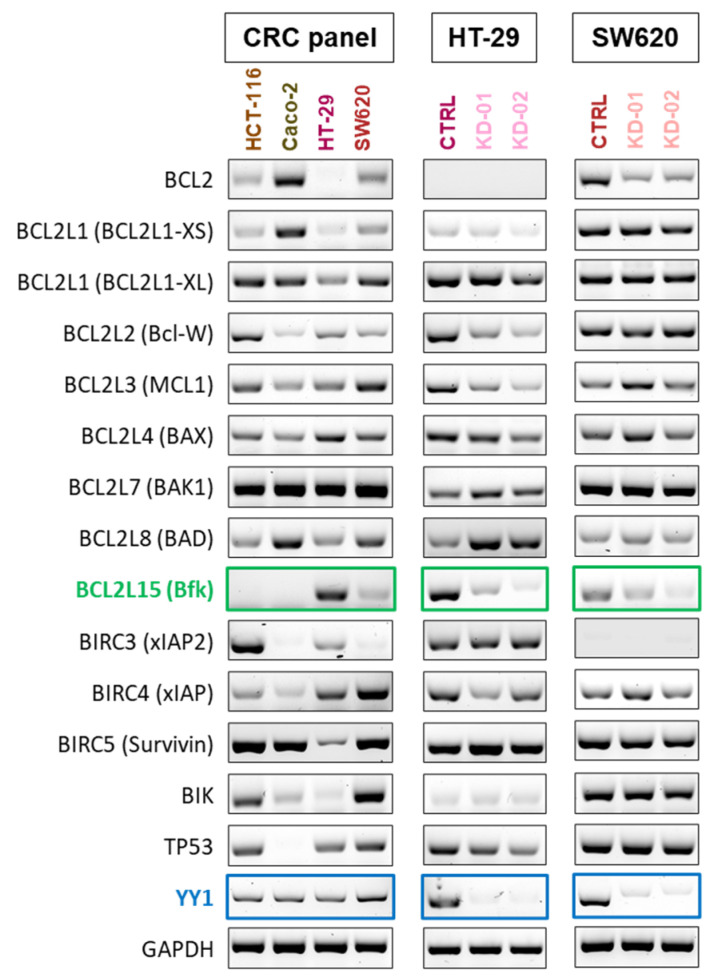
sq-RT-PCR analysis of YY1 putative transcriptional targets within the apoptotic pathway. Gel electrophoresis results. In CRC cells panel (HCT-116, Caco-2, HT-29 SW620); HT-29 (CTRL, KD-01, KD-02); SW620 (CTRL, KD-01, KD-02). *YY1* (blue) is expressed in all four cell lines. *BCL2L15* (green) is selectively expressed in HT-29 and SW620 cells and not detected in HCT-116 and Caco-2 cells. Upon *YY1* knock-down (blue boxes), *BCL2L15* is downregulated in both HT-29 and SW620 CRC cells (both KD-01 and KD-02, green boxes).

To identify possible transcriptional targets of YY1 in both HT-29 and SW620 cells, a list of previously identified YY1-putative targets was analyzed through sq-RT-PCR screening [23]. Figure 3 summarizes the results obtained for the four CRC cell lines (HCT-116, Caco-2, HT-29 and SW620 CTRL cells), as well as for HT-29 and SW620 CTRL, KD-01 and KD-02 clones. Strikingly, one of the genes analyzed, *BCL2L15*, was selectively expressed only in HT-29 and SW620 CRC responder cell lines and not detectable in both HCT-116 and Caco-2 non-responders, suggesting a difference among these two groups of CRC cancer cells (Figure 3 and Supplementary Figure S1).

If YY1 is regulating one or more putative target genes, their level of expression might be modulated in both HT-29 and SW620 CRC cells upon *YY1* silencing. The RT-PCR results showed that HT-29 and SW620 cells express differential basal levels of several transcripts (Figure 3, Supplementary Figures S2 and S3). Additionally, although some genes were found differentially modulated either in HT-29 or SW620 CRC cell lines upon *YY1*-KD, only one gene, the pro-apoptotic *BCL2L15*—selectively expressed by HT-29 and SW620—was strongly and significantly downregulated in both *YY1*-KD clones of both responder CRC cells (Figure 3, Supplementary Figures S2 and S3). This analysis demonstrated the existence of a positive correlation between *YY1* silencing and *BCL2L15* downregulation, as a common feature of both HT-29 and SW620 responders.

**Figure 4:**
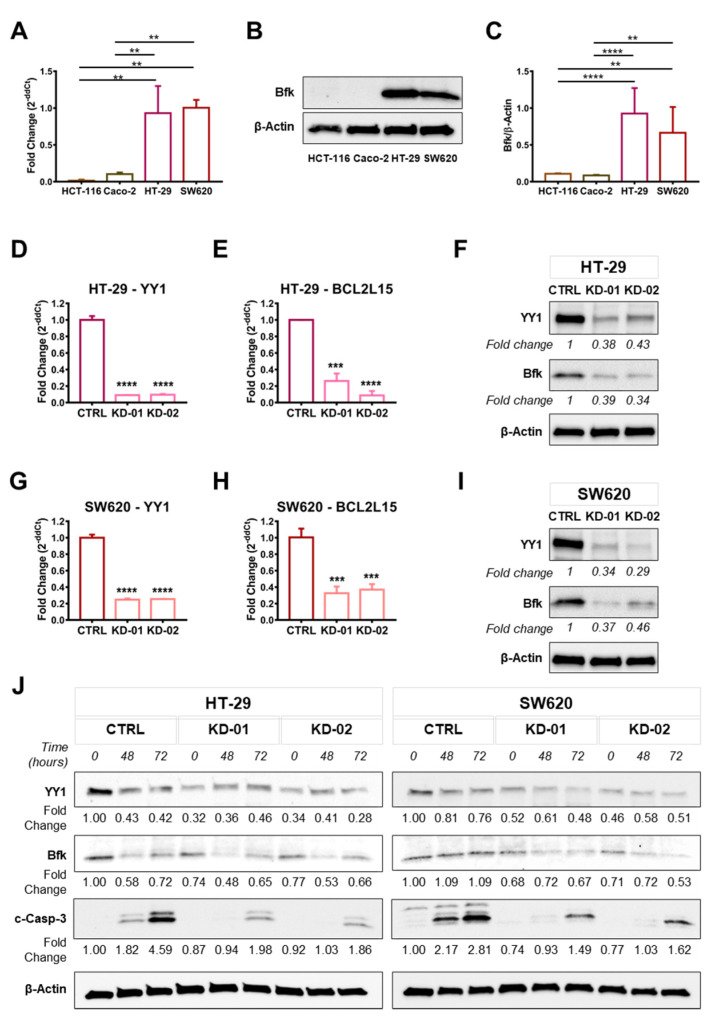
*BCL2L15*/Bfk is selectively expressed in HT-29 and SW620 CRC cells and it is downregulated upon *YY1* silencing. (**A**) q-RT-PCR analysis of *BCL2L15* expression in the four CRC cell lines (*GAPDH* used as housekeeping); data are expressed as 2^−ddCt^ and compared with SW620 normalized expression. (**B**) Immunoblot of Bfk (17 KDa) and β-Actin (42 KDa) proteins expression in the four CRC cell lines. (**C**) Densitometry analysis of Bfk expression (normalized to β-Actin). (**D**) q-RT-PCR analysis of *YY1* expression in HT-29 (CTRL, KD-01, KD-02), GAPDH used as housekeeping, data are expressed as 2^−ddCt^ compared to CTRL. (**E**) q-RT-PCR analysis of *BCL2L15* expression in HT-29 (CTRL, KD-01, KD-02), *GAPDH* used as housekeeping, data are expressed as 2^−ddCt^ compared to CTRL. (**F**) Immunoblot and densitometry of HT-29 (CTRL, KD-01, KD-02). Signal detected for YY1, Bfk and β-Actin. (**G**) q-RT-PCR analysis of *YY1* expression in SW620 (CTRL, KD-01, KD-02), *GAPDH* used as housekeeping, data are expressed as 2^−ddCt^ compared to CTRL. (**H**) q-RT-PCR analysis of *BCL2L15* expression in SW620 (CTRL, KD-01, KD-02), *GAPDH* used as housekeeping, data are expressed as 2^−ddCt^ compared to CTRL. (**I**) Immunoblot and densitometry of SW620 (CTRL, KD-01, KD-02). Signal detected for YY1, Bfk and β-Actin. (**J**) Immunoblot and densitometry of HT-29 and SW620 (CTRL, KD-01, KD-02) treated with 30µM 5-FU, from 0 to 72 h. Signal detected for YY1, Bfk, c-Casp-3 and β-Actin. Values are presented as Mean  ±  SD. ** *p* < 0.01; *** *p* < 0.001; **** *p* < 0.0001.

To further validate the screening results, q-RT-PCR and immunoblot analyses were performed. The results confirmed that *BCL2L15* mRNA (Figure 4A) and the corresponding Bfk protein (Figure 4B,C) were undetectable in both HCT-116 and Caco-2 non-responder CRC cells, while they were significantly detected in both HT-29 and SW620. Moreover, upon *YY1* silencing, both HT-29 and SW620 CRC showed a strong and significant downregulation of *BCL2L15*/Bfk, in both *YY1*-KD clones compared to their unsilenced CTRL (Figure 4D–I). As demonstrated by the immunoblot reported in Figure 4J, consistently with the decreased apoptotic response, in the *YY1*-KD HT-29 and SW620 cells it was observed a significantly stronger downregulation of Bfk protein following 72 h 5-FU treatment, within KD-01 and KD-02 cells compared with CTRL. Overall, the results reported in Figure 4 demonstrated that the pro-apoptotic factor *BCL2L15*/Bfk was selectively expressed in HT-29 and SW620, where, when *YY1* was KD, it was strongly downregulated and also faster reduced upon 5-FU-induced apoptosis.

### 2.4. YY1 and BCL2L15 Are Positive Diagnostic and Prognostic Markers in CRC Patients

To explore the nature of the association between YY1 and *BCL2L15*, ENCODE-deposited ChIP-Seq experiments were analyzed through the use of the SPP online resource. The analysis revealed that the promoter and enhancer regions of *BCL2L15* (from −10 kb to +10 kb around the TSS) were selectively bound by about forty different transcription factors, eighteen of which were identified in gastrointestinal epithelial cancer cells (Supplementary Figures S4 and S5 and Supplementary Table S2). Although two different transcription regulators belonging to the C_2_H_2_ Zinc finger family strongly bound the transcriptional regulatory region of *BCL2L15* (i.e., SP1 and KLF5), YY1 did not directly bind the *BCL2L15* promoter/enhancer, thus suggesting an indirect modulation.

Twelve independent whole human genome expression array datasets, generated with human CRC samples, were selected from the NCBI GEO Records (Supplementary Table S3). Supplementary Figure S6 and Supplementary Table S3 show the absence of any significant correlation between *YY1* and *BCL2L15* expression. In line with ChIP-Seq, the datasets correlation analyses also indicated that the association between YY1 and *BCL2L15* might be indirect.

**Figure 5:**
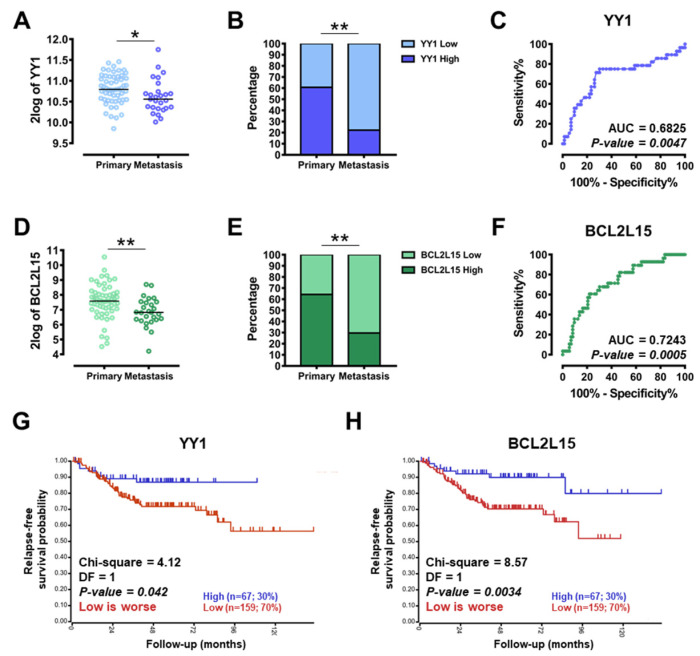
Diagnostic and prognostic value of *YY1* and *BCL2L15* in CRC patients’ cohorts. (**A**) GSE28702, dot plots with median of *YY1* 2log expression in CRC samples primary and metastasis. (**B**) GSE28702, Fisher’s exact test, data represented as *YY1* low vs. high percentage expression in CRC samples, primary vs. metastasis. (**C**) GSE28702, receiver operating characteristics (ROC) analysis of *YY1* expression in CRC samples primary vs. metastasis. (**D**) GSE28702, dot plots with median of *BCL2L15* 2log expression in CRC samples primary and metastasis. (**E**) GSE28702, Fisher’s exact test, data represented as *BCL2L15* low vs. high percentage expression in CRC samples primary vs. metastasis. (**F**) GSE28702, ROC analysis of *BCL2L15* expression in CRC samples primary vs. metastasis. (**G**) GSE14333, Kaplan–Meier analysis of relapse-free survival correlated with *YY1* expression (high:low = 30%:70%). (**H**) GSE14333, Kaplan–Meier analysis of relapse-free survival correlated with *BCL2L15* expression (high:low = 30%:70%). * *p* < 0.05; ** *p*  <  0.01.

To assess the potential diagnostic and prognostic value of *YY1* and *BCL2L15* tumor-suppressor genes, relevant CRC datasets were analyzed for features linked with CRC aggressiveness. In particular, the expression of both *YY1* and *BCL2L15* was analyzed in the GSE28702 dataset (Yagi, 83 CRC samples), consisting of 56 primary tumors and 27 metastatic lesions (23 from liver, 1 from lung and 3 from peritoneum), obtained from patients before any chemotherapy or radiotherapy. Importantly, the expression of both *YY1* and *BCL2L15* was significantly lower in metastases compared with primary tumors (respectively *p* = 0.0422 and *p* = 0.0077; Figure 5A,D). As reported in Figure 5B,E, the contingency analysis through Fisher’s exact test evidenced an extremely significant difference in both *YY1* and *BCL2L15* expression, with a 78% of *YY1*-low expressing samples and a 70% *BCL2L15*-low expressing samples within the metastasis subgroup. Consistently, the ROC curves in Figure 5C and 5F showed an area under the curve (AUC) of 0.68 for *YY1* (*p* = 0.0047) and 0.72 for *BCL2L15* (*p* = 0.0005). These high and significant AUC performances suggested that both *YY1* and *BCL2L15* expression levels can be considered as diagnostic discriminators between primary—less aggressive, and metastasis—more aggressive, subgroups in CRC-affected subjects.

To additionally validate the potential prognostic role of both *YY1* and *BCL2L15*, the GSE14333 dataset (Sieber, 290 surgically resected primary CRCs) was analyzed through the R2 function “Kaplan–Meier by gene expression”. As evidenced by the results reported in Figure 5G,H, when the expression of both *YY1* and *BCL2L15* was split into low- vs. high-expressing subgroups (70% vs. 30%), the relapse-free survival probability curves showed a significantly lower chance of survival within the low-expressing groups for both genes (Chi-squared 4.12 and 8.57 and *p*-value 0.0420 and 0.0034, respectively for *YY1* and *BCL2L15*). These results suggested that both *YY1* and *BCL2L15* might represent positive prognostic factors, in line with their proposed role as tumor-suppressors. In summary, the dataset analyses reported in Figure 5 demonstrated that *YY1*, as well as *BCL2L15* expression levels, may be useful biomarkers for diagnosis and prognosis in CRC patients.

### 2.5. YY1 and BCL2L15 Expression Is Correlated with Selected CRC Molecular Subtypes and Specific Single-Cell Hierarchical Clustering

**Figure 6:**
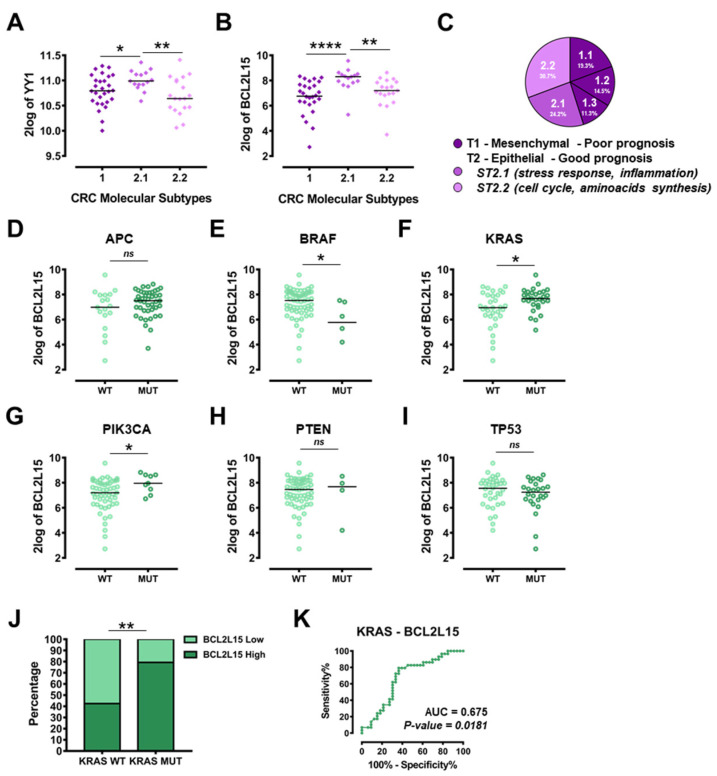
Correlation of *YY1* and *BCL2L15* expression with CRC molecular subtypes and mutational status. (**A**) GSE35896, dot plots with median of *YY1* 2log expression in CRC samples (T1, ST2.1, ST2.2). (**B**) GSE35896, dot plots with median of *BCL2L15* 2log expression in CRC samples (T1, ST2.1, ST2.2). (**C**) GSE35896, cake plot of CRC samples types and subtypes (percentage of the total). GSE35896, dot plots with median of *BCL2L15* 2log expression in CRC samples of: (**D**) APC (WT vs. MUT). (**E**) BRAF (WT vs. MUT). (**F**) KRAS (WT vs. MUT). (**G**) PIK3CA (WT vs. MUT). (**H**) PTEN (WT vs. MUT). (**I**) TP53 (WT vs. MUT). (**J**) GSE35896, Fisher’s exact test, data represented as *BCL2L15* low vs. high percentage expression in CRC samples, KRAS WT vs. KRAS MUT. (**K**) ROC analysis of *BCL2L15* expression in CRC samples KRAS WT vs. KRAS MUT. * *p* < 0.05; ** *p*  <  0.01; **** *p*  <  0.0001; n.s. = not significant.

Given the intrinsic genetic heterogeneity of CRCs, the relative expression of both *YY1* and *BCL2L15* was analyzed within the GSE35896 dataset (Wessels, 62 primary CRC samples), with samples stratified based on their molecular types associated with a gene expression signature of epithelial-mesenchymal transition (EMT; Figure 6C). In particular, Type 1 (T1) is associated with a mesenchymal signature and a poorer prognosis, while Type 2 (T2) is linked with an epithelial signature and a better prognosis. T2 can be further divided into Subtypes 2.1 and 2.2 (ST2.1 and ST2.2). While the ST2.1 is associated with the overactivation of pathways involved in pro-inflammatory and stress-related responses, the ST2.2 is linked with the overexpression of pathways regulating the cell cycle (Figure 6C) [24]. Importantly, as reported in Figure 6A,B, the expression of both *YY1* and *BCL2L15* was significantly higher in the ST2.1, which represents the epithelial-like, pro-inflammatory molecular subgroup with a better prognosis. These results suggested positive diagnostic and prognostic roles for both *YY1* and *BCL2L15*, in line with the above-reported findings.

The same GSE35896 dataset allowed the stratification of CRC patients based on their specific mutational background within key tumor-driver genes. Interestingly *YY1* expression was not statistically different in any of the stratifications, suggesting that *YY1* expression was not associated with the specific mutational status for none of the genes analyzed (Supplementary Figure S7). On the contrary, as shown in Figure 6D–I, the expression of *BCL2L15* varied significantly, being higher in *KRAS* and *PIKC3A* mutant (MUT) compared to wild-type (WT) specimens (respectively *p* = 0.0175 and *p* = 0.0206), and significantly lower in *BRAF* MUT compared with WT (*p* = 0.0137). Among the mutations analyzed, *KRAS* was the only one associated with a diagnostic predictive power for *BCL2L15*, as the chi-squared test was highly significant (*p* = 0.0043), with 79% of *BCL2L15*-high expressing subjects in the MUT subgroup (Figure 6J). Accordingly, the AUC calculated within the ROC curve showed a value of 0.675 (*p* = 0.0181; Figure 6K). In summary, both *YY1* and *BCL2L15* expressions were significantly higher in the ST2.1 subgroup (epithelial, pro-inflammatory, low-aggressive, better prognosis), while only *BCL2L15* expression was selectively modulated when patients were stratified based on their specific mutational background.

**Figure 7:**
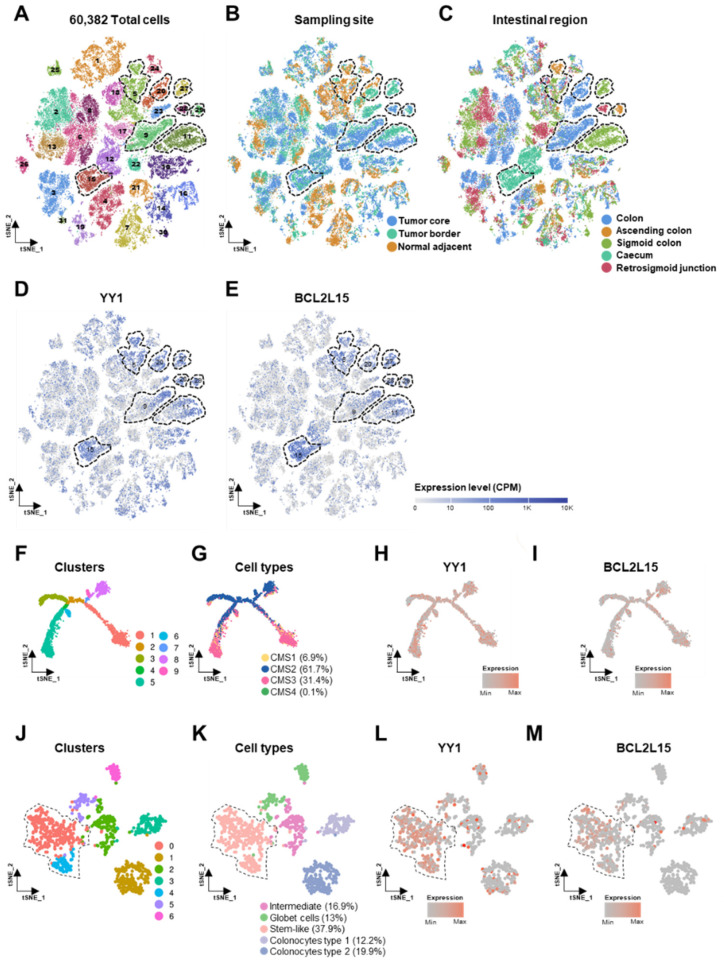
Correlation of *YY1* and *BCL2L15* expression with single-CRC-cells features in GSE132465 and GSE144735 datasets. (**A**) t-distributed stochastic neighbor embedding (t-SNE) plot of 60,382 CRC total cells (clusters). (**B**) t-SNE plot of CRC total cells (sampling site). (**C**) t-SNE plot of CRC total cells (intestinal region). (**D**) t-SNE plot of CRC total cells (YY1 expression). (**E**) t-SNE plot of CRC total cells (*BCL2L15* expression). Expression level as counts per million reads mapped (CPM). (**F**) t-SNE plot of 17,469 tumor epithelial cells (clusters). (**G**) t-SNE plot of tumor epithelial cells (CMS signatures). (**H**) t-SNE plot of tumor epithelial cells (*YY1* expression). (**I**) t-SNE plot of tumor epithelial cells (*BCL2L15* expression). (**J**) t-SNE plot of 1070 normal epithelial cells (clusters). (**K**) t-SNE plot of normal epithelial cells (cell types). (**L**) t-SNE plot of normal epithelial cells (*YY1* expression). (**M**) t-SNE plot of normal epithelial cells (*BCL2L15* expression). Dotted lines highlight areas of *YY1* and *BCL2L15* high expression.

To further assess the specific expression pattern of *YY1* and *BCL2L15* at the single-cell level, two datasets composed of a total of 60,382 CRC single-cell transcriptomes, deriving from 29 patients (GSE132465 and GSE144735), were analyzed through the use of two publicly available analysis tools. The CRC cells were stratified based on their different hierarchical clusters, sampling site and colorectal distribution, as shown by the t-distributed stochastic neighbor embedding (t-SNE) plots in Figure 7A–C. The expression pattern of *YY1* and *BCL2L15* were heterogeneous within the different sampling sites and intestinal regions (Figure 7D,E). Both *YY1* and *BCL2L15* expression levels were selectively enriched in 7 out of the 31 hierarchical clusters, with overlapping areas in the relative tSNE plots (dotted line areas). In particular, clusters 9, 11, 15, 27, 28 and 29 (composed of tumor cells deriving from tumor core and border), plus clusters 5 and 20 (additionally enriched in normal adjacent cells) showed high *YY1* and *BC2L15* co-expression (Figure 7A–E).

By selectively looking into the epithelial tumor cellular component and stratifying the hierarchical clusters based on the CMS [4], it was possible to observe a heterogeneous expression of both *YY1* and *BCL2L15* (Figure 7F–I). On the other hand, in normal adjacent epithelial cells, the distribution of *YY1* and *BCL2L15* was preferentially enriched in the stem-like subgroup (clusters 0 and 4), overall representing 38% of the whole normal epithelial cells (Figure 7J–M). Strikingly, the tSNE plots of *YY1* and *BCL2L15* expressions in other main non-tumoral cellular components (stromal cells, myeloid, T and B cells) showed a selectively higher and heterogenous expression of *YY1*. Contrariwise, *BCL2L15* was very low-represented (Supplementary Figure S8).

Overall, the single-CRC cell transcriptome sequencing analyses demonstrated that *YY1* and *BCL2L15* co-expression was enriched in selected clones of whole-CRC cellular clusters. Interestingly, *YY1* and *BCL2L15* were greatly expressed within the tumoral epithelial cells and the stem-like adjacent normal epithelial cells. The other kinds of non-tumoral cells, including stromal cells, myeloid, T and B cells, expressed heterogenous and detectable levels of *YY1*, whereas *BCL2L15* was found to be underrepresented.

## 3. Discussion

CRC is characterized by relevant genomic instability and concurrent tumor heterogeneity. In order to study the role of YY1 in CRC, a panel of four genetically different CRC cell lines was selected (Supplementary Table S1). In this study, it was found that two out of the four cell lines tested, the HT-29 and SW620, when silenced for *YY1*, were less sensitive to 5-FU-induced cytotoxicity and therefore protected against 5-FU-mediated cell death. Even if isolated from different patients, the responder HT-29 and SW620 cells are both Dukes’ C adenocarcinomas. Additionally, both cells have a pro-tumorigenic “gain-of-function” TP53 mutation (R273H) and a non-sense *APC* mutation, while *CTNNB1* is WT (Supplementary Table S1) [25,26,27,28]. Regarding *YY1* expression, one study recently reported that *YY1* has one copy loss in HT-29, but not in HCT-116, Caco-2 or SW620 CRC cells [26]. This result is in line with the immunoblot findings, where the overall detected YY1 protein level was significantly lower in HT-29 (Figure 1B).

YY1 is a multifaceted factor in oncology and its function mostly depends on the molecular environment and specific tumor type [29]. Indeed, the involvement of YY1 in cancer development and progression depends on different genetic and epigenetic factors able to modulate its expression [30,31,32]. Additionally, YY1 might interact directly with the target gene-regulatory regions, but also bind a wide range of co-factors, histones modifying enzymes and PcG proteins [9,33]. In CRC, YY1 is prevalently considered pro-tumorigenic, although the data, especially from patients, are few and several incongruences have been reported [10]. To the best of our knowledge, this is the first report which clearly demonstrated a tumor-suppressive role for YY1 in CRC, and, in particular, its positive role in chemosensitivity (as *YY1* higher levels were associated with greater CRC cellular death in responder cell lines).

The viability results in CRC cells corroborated the involvement of the apoptotic pathway, as *YY1* modulation affects the apoptotic response of both HT-29 and SW620 cells (Figure 2). Hence, the RT-PCR screening allowed the identification of CRC cell-type-specific footprints, in terms of apoptotic genes basal expression, as well as their modulation upon *YY1*-KD. Although *YY1* KD affected several and different apoptosis regulator genes in HT-29 and SW620 responder cells, one gene was consistently downregulated in both YY1-KD clones of both HT-29 and SW620: *BCL2L15*. This result was robustly confirmed both at transcript and protein levels (Figure 4).

*BCL2L15* belongs to the Bcl-2 family of apoptotic gene regulators and encodes for Bfk, preferentially expressed by normal gastrointestinal epithelial cells and previously found downregulated in colon tissues from CRC patients [34]. It was demonstrated that colon cells overexpressing *BCL2L15*/Bfk undergo apoptosis, although it is controversial whether Bfk is activated following effector caspases cleavage or not [35,36]. The fact that this pro-apoptotic factor was downregulated in association with *YY1* silencing, is consistent with the functional results hereby presented in terms of apoptotic response, which was lower in *YY1*-KD responder CRC cells. Interestingly, *BCL2L15*/Bfk was selectively detected in HT-29 and SW620, but not in HCT-116 and Caco-2 cells, in line with previously reported findings [34]. Consistently, amongst the cell lines tested, only HT-29 and SW620 differentially responded to 5-FU in terms of apoptotic response upon *YY1* silencing (Figure 4J).

The analysis of ChIP-seq deposited data failed to support any direct binding of YY1 within the *BCL2L15* transcriptional regulatory region. Moreover, *YY1* and *BCL2L15* gene expression analyses in twelve different CRC datasets revealed the absence of any significant correlation. Overall, the results suggested that YY1 might indirectly regulate the expression of *BCL2L15*. Future studies will be needed to characterize the nature of the indirect interaction hereby postulated. Indeed, a recent study showed that *BCL2L15* may be a transcriptional target of the PROX1 homeobox transcription factor, and that PROX1-mediated repression of *BCL2L15* is important for the survival of CRC cells subjected to metabolic stress [37]. Additionally, PROX1 is upregulated in mice embryos KO for *YY1*, thus suggesting that YY1 may negatively regulate PROX1 expression [38]. Alternatively, YY1 could recruit enzymes able in turn to modify the chromatin (es., EZH2, HDAC) and, hence, orchestrate changes in the epigenetic status of the *BCL2L15* gene, as it was observed in other cancer and non-cancer models [11,39,40]. YY1 might also bind chromatin sites far from the regulatory region of *BCL2L15* and, at a far distance, modulate the *BCL2L15* locus rearrangement [41,42].

The tumor-suppressive role found for both YY1 and *BCL2L15*/Bfk in CRC cells was further validated in patients, by analyzing relevant CRC datasets. The analysis of *YY1* and *BCL2L15* expression following CRC samples stratification based on cancer aggressiveness (i.e., metastasis vs. primary, mesenchymal vs. epithelial) was demonstrated to be a significant discriminator. Importantly, in GSE28702 the expression of both *YY1* and *BCL2L15* was significantly lower in metastatic than primary CRC samples (Figure 5). Additionally, in GSE35896 the expression levels of both *YY1* and *BCL2L15* were significantly upregulated in the less aggressive and pro-inflammatory epithelial-like subtype ST2.1 (Figure 6) [24]. Moreover, within the same dataset, upon sample stratification based on mutational background, *BCL2L15* (but not *YY1*) was significantly upregulated in samples carrying *PIK3CA* or *KRAS* mutation and downregulated in samples carrying *BRAF* mutation. Interestingly, the expression levels of *BCL2L15* showed a diagnostic relevance within CRC patients specifically carrying the *KRAS* mutation.

To further explore the specific expression pattern of both *YY1* and *BCL2L15*, two single-cell-sequencing CRC datasets were analyzed (GSE132465, GSE144735). Intriguingly, *YY1* and *BCL2L15* genes were found highly and selectively co-expressed, in 7 out of 31 whole CRC cell clusters. Additionally, the expression of such genes in a selected population of cells was explored. The expression of both *YY1* and *BCL2L15* was higher and diffused in CRC epithelial tumor cells, as well as in a specific subset of normal (non-tumoral) adjacent epithelial cells with stem-like features (Figure 7). This latter result is in line with the observed role played by YY1 and Bfk in gut development, specifically in the stem cell niche [37,43,44]. In vivo studies demonstrated that when YY1 is knocked out in the gut, mice develop defective villi, where the crypts-resident stem cells gradually lose their renewal capacity [43,44]. Importantly, within the other non-tumor cells (stromal, myeloid, B and T cells) only *YY1* was expressed, while *BCL2L15* was sparsely detected (Supplementary Figure S8). This latter observation highlights a potential additional role for YY1 in normal non-transformed peritumoral cells, especially within the immune system, as suggested in other contexts [45,46].

Finally, the analysis of survival data (GSE14333) corroborated that CRC patients that strongly express both *YY1* and *BCL2L15* have a significantly higher chance of relapse-free survival (Figure 5). In conclusion, our study uncovered the tumor-suppressive role of YY1 and *BCL2L15*, and their potential value as biomarkers useful in both diagnosis and prognosis of CRC patients. In the future, a validation of these novel findings in larger cohorts of patients is needed to gain further robustness.

## 4. Materials and Methods

### 4.1. Cell Lines and Culture

HCT-116, Caco-2, HT-29 and SW620 human CRC cell lines (Supplementary Table S1) were purchased from the American Type Culture Collection (Manassas, VA, USA) [25,26,27]. HCT-116, HT-29 and SW620 cells were grown in RPMI 1640, while Caco-2 in MEM. 293-LinX-A packaging cell line (kindly provided by Dr. Roberta Maestro, Aviano, Italy) was cultured in DMEM. All culture media (Sigma-Aldrich, St. Louis, MO, USA) were supplemented with 2 mmol/L L-glutamine, 100 IU penicillin, 100 μg/mL streptomycin and 10% heat-inactivated Fetal Bovine Serum (Sigma-Aldrich, St. Louis, MO, USA). Cells were maintained in a humidified, 37 °C and 5% CO_2_ incubator and used within 15 passages after thawing. Mycoplasma absence was assessed by PCR Assay.

### 4.2. Generation of CRC Cells Constitutively Silenced for YY1

To generate cells CRC cells constitutively silenced for *YY1*, specific retroviral plasmid vectors were employed. pSMP-YY1_1 and pSMP-YY1_2 and pSMP-Luc (non-silencing control), were generated by George Daley and deposited in Addgene plasmid bank (respectively addgene-36357, addgene-36358; addgene-36394, Addgene, Watertown, MA, USA; Figure 1C) [47]. Generation of retroviral particles, transduction and clonal selection of target CRC cells (with Puromycin 1 µg/mL final, Sigma-Aldrich, St. Louis, MO, USA) were performed according to the published protocol [48,49,50].

### 4.3. Cell Viability Assays

The 3-(4,5-Dimethylthiazol-2-yl)-2,5-diphenyl tetrazolium bromide (MTT, Sigma-Aldrich, St. Louis, MO, USA) assay was used to assess cellular viability. For anti-cancer treatments, cells were treated with 5-Fluorouracil (5-FU; Sigma-Aldrich, St. Louis, MO, USA), with concentrations ranging from 5.0 × 10^−4^ to 7.6 × 10^−9^ M for 72 h. The cells were assessed for their viability by adding 0.5 µg/mL MTT per well. Insoluble formazan crystals were dissolved by adding an acid-isopropanol stop solution (0.04 N HCl). Absorbance was measured at 610 nm, using the Tecan-Sunrise microplate reader (Tecan, Männedorf, Switzerland). For trypan blue count, cellular samples were mixed 1:1 with 0.4% Trypan Blue (Thermo Fisher Scientific, Waltham, MA, USA). Cells permeable to Trypan Blue were counted as dead. Counts were performed in triplicate by using a Bürker chamber and the Eclipse Ts2 inverted microscope (Nikon, Melville, NY, USA).

### 4.4. Total RNA Extraction, cDNA Synthesis and Semiquantitative and Quantitative RT-PCR Analyses

For total RNA extraction, up to 3 × 10^6^ cells were harvested and total RNA was isolated using GeneJET RNA Purification Kit (Thermo Fisher Scientific, Waltham, MA, USA). For cDNA synthesis, 3 µg of the total RNA was reverse-transcribed with Super-Script IV Reverse Transcriptase (Thermo Fisher Scientific, Waltham, MA, USA).

The template cDNA was amplified using the primer pairs designed using the Primer-Blast priming designing tool from NCBI (Supplementary Table S4) [51]. The expression levels of target genes were normalized to the averaged expression levels of the human *GAPDH* housekeeping gene. DreamTaq Green PCR Master Mix was used for semi-quantitative RT-PCR (sq-RT-PCR), while Luminaris Color HiGreen qPCR Master Mix, high ROX for quantitative RT-qPCR (q-RT-PCR; both Thermo Fisher Scientific, Waltham, MA, USA). 7300 Real-Time PCR System was employed to detect cDNA amplification (Thermo Fisher Scientific, Waltham, MA, USA).

### 4.5. Protein Lysates Preparation, Quantification and Immunoblot Analyses

For protein extraction, up to 5 × 10^6^ cells were harvested. The collected cells were lysed using nonidet-P40 buffer (Thermo Fisher Scientific, Waltham, MA, USA) supplemented with protease and phosphatase inhibitors (Roche Diagnostics, Indianapolis, IN, USA). Protein concentration was determined with Bradford assay (Bio-Rad Laboratories, Hercules, CA, USA). Protein samples were separated using Mini-PROTEAN precast gels and gel–electrophoresis system; protein gels were transferred using TransBlot Turbo transfer system (Bio-Rad Laboratories, Hercules, CA, USA). Nitrocellulose membranes were blocked with 5% of non-fat dry milk diluted in TBS-T buffer (0.1% Tween 20, 20 mM Tris–HCl pH 7.6, 137 mM NaCl). Immunoblotting analysis was performed using the antibodies and dilutions reported in Supplementary Table S5. Enhanced chemiluminescence signals were acquired with the ChemiDoc Touch Imaging System (Bio-Rad Laboratories, Hercules, CA, USA). The anti-YY1 antibody was tested for its mono-specificity. FLAG-tagged human-recombinant YY2 (hr-YY2, RC223433, OriGene Technologies, Rockville, MD, USA) was used as the internal control. The immunoblot results demonstrated that YY1 protein, and not hrYY2, was specifically recognized by the anti-YY1 antibody (Supplementary Figure S9).

### 4.6. Bioinformatic Analyses

The following bioinformatic portals with data repositories were used: ENCODE, Gene Expression Omnibus (GEO) and European Bioinformatics Institute (EMBL-EBI) [52,53,54]. The Transcription Factor ChIP-seq experiments deposited in ENCODE were analyzed using the publicly available Signaling Pathways Project (SPP) online resource at the query interface Ominer [55].

The CRC GEO DataSets of gene expression analyzed in this study are reported in Supplementary Table S3 (Affymetrix HGU133 P2.0 expression arrays; Log2 gene expression values calculated using MAS5.0 algorithm). To establish the expression levels of *YY1* and *BCL2L15,* the datasets obtained from GEO DataSets were analyzed by using the R2 Genomics Analysis and Visualization Platform [56].

*YY1* and *BCL2L15* gene expression and clusters distribution in the single CRC cell RNA-Seq data generated by Lee et al. [57] and deposited in the publicly available GSE132465 and GSE144735 datasets (Illumina), were analyzed by using the following two interface platforms: Cambridge Portal of the Human Cell Atlas (EMBL-EBI) and user-friendly InteRface tool to Explore Cell Atlas (URECA, Korean Bioinformation Centre) [58,59].

### 4.7. Statistical Analyses

Statistical analyses were performed using GraphPad Prism version 7.0 for Windows (GraphPad Software, La Jolla, CA, USA). Results were presented as average ± standard deviation (SD) or as a median. Single parameter comparisons between two groups were conducted using two-tailed unpaired Student’s *t*-test (parametric data) or Mann–Whitney’s U-test (non-parametric data). Single parameter comparisons between three or more groups were performed using one-way analysis of variance (ANOVA) with Tukey’s or Dunnett’s multiple comparison test (parametric data) or Kruskal–Wallis H-test (non-parametric data). Multiple parameter comparisons between two groups were performed using two-way ANOVA with Tukey’s multiple comparison test. The normalized expression value distribution of both *YY1* and *BCL2L15* in CRC GEO DataSets was evaluated with a D’Agostino and Pearson normality test. The contingency analyses of the relevant datasets were performed by using the chi-squared test or Fisher’s exact test. The ROC curve analyses and AUC calculations were used to predict both *YY1* and *BCL2L15* diagnostic relevance. Differences were considered significant with *p*-values < 0.05; being: * *p* < 0.05; ** *p* < 0.01; *** *p* < 0.001; **** *p* < 0.0001.

## Data Availability

Not applicable.

## Supplementary Materials

The following are available online at https://www.mdpi.com/article/10.3390/ijms22168481/s1.

## References

[1] Bray F., Ferlay J., Soerjomataram I., Siegel R.L., Torre L.A., Jemal A. (2018). Global cancer statistics 2018: GLOBOCAN estimates of incidence and mortality worldwide for 36 cancers in 185 countries. CA Cancer J. Clin..

[2] Arnold M., Sierra M.S., Laversanne M., Soerjomataram I., Jemal A., Bray F. (2017). Global patterns and trends in colorectal cancer incidence and mortality. Gut.

[3] Molinari C., Marisi G., Passardi A., Matteucci L., De Maio G., Ulivi P. (2018). Heterogeneity in Colorectal Cancer: A Challenge for Personalized Medicine?. Int. J. Mol. Sci..

[4] Guinney J., Dienstmann R., Wang X., de Reyniès A., Schlicker A., Soneson C., Marisa L., Roepman P., Nyamundanda G., Angelino P. (2015). The consensus molecular subtypes of colorectal cancer. Nat. Med..

[5] Menter D.G., Davis J.S., Broom B.M., Overman M.J., Morris J., Kopetz S. (2019). Back to the Colorectal Cancer Consensus Molecular Subtype Future. Curr. Gastroenterol. Rep..

[6] Vogelstein B., Papadopoulos N., Velculescu V.E., Zhou S., Diaz L.A., Kinzler K.W. (2013). Cancer genome landscapes. Science.

[7] Patel J.N., Fong M.K., Jagosky M. (2019). Colorectal Cancer Biomarkers in the Era of Personalized Medicine. J. Pers. Med..

[8] Gordon S., Akopyan G., Garban H., Bonavida B. (2006). Transcription factor YY1: Structure, function, and therapeutic implications in cancer biology. Oncogene.

[9] Meliala I.T.S., Hosea R., Kasim V., Wu S. (2020). The biological implications of Yin Yang 1 in the hallmarks of cancer. Theranostics.

[10] Sarvagalla S., Kolapalli S.P., Vallabhapurapu S. (2019). The Two Sides of YY1 in Cancer: A Friend and a Foe. Front. Oncol..

[11] Tang W., Zhou W., Xiang L., Wu X., Zhang P., Wang J.J.J.J., Liu G., Zhang W., Peng Y., Huang X. (2019). The p300/YY1/miR-500a-5p/HDAC2 signalling axis regulates cell proliferation in human colorectal cancer. Nat. Commun..

[12] Chinnappan D., Xiao D., Ratnasari A., Andry C., King T.C., Weber H.C. (2009). Transcription factor YY1 expression in human gastrointestinal cancer cells. Int. J. Oncol..

[13] Sui Y., Wu T., Li F., Wang F., Cai Y., Jin J. (2019). YY1/BCCIP Coordinately Regulates P53-Responsive Element (p53RE)-Mediated Transactivation of p21Waf1/Cip1. Int. J. Mol. Sci..

[14] Fang Z., Yang H., Chen D., Shi X., Wang Q., Gong C., Xu X., Liu H., Lin M., Lin J. (2019). YY1 promotes colorectal cancer proliferation through the miR-526b-3p/E2F1 axis. Am. J. Cancer Res..

[15] Zhang L., Dong X., Yan B., Yu W., Shan L. (2020). CircAGFG1 drives metastasis and stemness in colorectal cancer by modulating YY1/CTNNB1. Cell Death Dis..

[16] Ye Y., Gu B., Wang Y., Shen S., Huang W. (2019). YY1-Induced Upregulation of Long Noncoding RNA ARAP1-AS1 Promotes Cell Migration and Invasion in Colorectal Cancer Through the Wnt/β-Catenin Signaling Pathway. Cancer Biother. Radiopharm..

[17] Zhu G., Qian M., Lu L., Chen Y., Zhang X., Wu Q., Liu Y., Bian Z., Yang Y., Guo S. (2019). O-GlcNAcylation of YY1 stimulates tumorigenesis in colorectal cancer cells by targeting SLC22A15 and AANAT. Carcinogenesis.

[18] Yu J., Wang F., Zhang J., Li J., Chen X., Han G. (2020). LINC00667/miR-449b-5p/YY1 axis promotes cell proliferation and migration in colorectal cancer. Cancer Cell Int..

[19] Yokoyama N.N., Pate K.T., Sprowl S., Waterman M.L. (2010). A role for YY1 in repression of dominant negative LEF-1 expression in colon cancer. Nucleic Acids Res..

[20] Sui Y., Li F., Wu T., Ding J., Lu Z., Wang L., Yang Y., Wang F., Zhao L., Zhu H. (2018). BCCIP binds to and activates its promoter in a YY1-dependent fashion in HCT116 cells. FEBS J..

[21] Su J., Sui Y., Ding J., Li F., Shen S., Yang Y., Lu Z., Wang F., Cao L., Liu X. (2016). Human INO80/YY1 chromatin remodeling complex transcriptionally regulates the BRCA2- and CDKN1A-interacting protein (BCCIP) in cells. Protein Cell.

[22] LIU X., CAO L., NI J., LIU N., ZHAO X., WANG Y., ZHU L., WANG L., WANG J., YUE Y. (2013). Differential BCCIP gene expression in primary human ovarian cancer, renal cell carcinoma and colorectal cancer tissues. Int. J. Oncol..

[23] Vivarelli S., Falzone L., Ligresti G., Candido S., Garozzo A., Magro G.G., Bonavida B., Libra M. (2020). Role of the Transcription Factor Yin Yang 1 and Its Selectively Identified Target Survivin in High-Grade B-Cells non-Hodgkin Lymphomas: Potential Diagnostic and Therapeutic Targets. Int. J. Mol. Sci..

[24] Schlicker A., Beran G., Chresta C.M., McWalter G., Pritchard A., Weston S., Runswick S., Davenport S., Heathcote K., Castro D.A. (2012). Subtypes of primary colorectal tumors correlate with response to targeted treatment in colorectal cell lines. BMC Med. Genom..

[25] Ahmed D., Eide P.W., Eilertsen I.A., Danielsen S.A., Eknæs M., Hektoen M., Lind G.E., Lothe R.A. (2013). Epigenetic and genetic features of 24 colon cancer cell lines. Oncogenesis.

[26] Berg K.C.G., Eide P.W., Eilertsen I.A., Johannessen B., Bruun J., Danielsen S.A., Bjørnslett M., Meza-Zepeda L.A., Eknæs M., Lind G.E. (2017). Multi-omics of 34 colorectal cancer cell lines—A resource for biomedical studies. Mol. Cancer.

[27] Mouradov D., Sloggett C., Jorissen R.N., Love C.G., Li S., Burgess A.W., Arango D., Strausberg R.L., Buchanan D., Wormald S. (2014). Colorectal cancer cell lines are representative models of the main molecular subtypes of primary cancer. Cancer Res..

[28] Ronen J., Hayat S., Akalin A. (2019). Evaluation of colorectal cancer subtypes and cell lines using deep learning. Life Sci. Alliance.

[29] De Nigris F., Zanella L., Cacciatore F., De Chiara A., Fazioli F., Chiappetta G., Apice G., Infante T., Monaco M., Rossiello R. (2011). YY1 overexpression is associated with poor prognosis and metastasis-free survival in patients suffering osteosarcoma. BMC Cancer.

[30] Terreri S., Durso M., Colonna V., Romanelli A., Terracciano D., Ferro M., Perdonà S., Castaldo L., Febbraio F., de Nigris F. (2016). New Cross-Talk Layer between Ultraconserved Non-Coding RNAs, MicroRNAs and Polycomb Protein YY1 in Bladder Cancer. Genes.

[31] Infante T., Mancini F.P., Lanza A., Soricelli A., de Nigris F., Napoli C. (2015). Polycomb YY1 is a critical interface between epigenetic code and miRNA machinery after exposure to hypoxia in malignancy. Biochim. Biophys. Acta-Mol. Cell Res..

[32] Hafsi S., Candido S., Maestro R., Falzone L., Soua Z., Bonavida B., Spandidos D.A., Libra M. (2016). Correlation between the overexpression of Yin Yang 1 and the expression levels of miRNAs in Burkitt’s lymphoma: A computational study. Oncol. Lett..

[33] De Nigris F., Crudele V., Giovane A., Casamassimi A., Giordano A., Garban H.J., Cacciatore F., Pentimalli F., Marquez-Garban D.C., Petrillo A. (2010). CXCR4/YY1 inhibition impairs VEGF network and angiogenesis during malignancy. Proc. Natl. Acad. Sci. USA.

[34] Dempsey C.E., Dive C., Fletcher D.J., Barnes F.A., Lobo A., Bingle C.D., Whyte M.K.B., Renshaw S.A. (2005). Expression of pro-apoptotic Bfk isoforms reduces during malignant transformation in the human gastrointestinal tract. FEBS Lett..

[35] Coultas L., Pellegrini M., Visvader J.E., Lindeman G.J., Chen L., Adams J.M., Huang D.C.S., Strasser A. (2003). Bfk: A novel weakly proapoptotic member of the Bcl-2 protein family with a BH3 and a BH2 region. Cell Death Differ..

[36] Ozören N., Inohara N., Núñez G. (2009). A putative role for human BFK in DNA damage-induced apoptosis. Biotechnol. J..

[37] Ragusa S., Cheng J., Ivanov K.I., Zangger N., Ceteci F., Bernier-Latmani J., Milatos S., Joseph J.-M., Tercier S., Bouzourene H. (2014). PROX1 promotes metabolic adaptation and fuels outgrowth of Wnt(high) metastatic colon cancer cells. Cell Rep..

[38] Affar E.B., Gay F., Shi Y., Liu H., Huarte M., Wu S., Collins T., Li E. (2006). Essential dosage-dependent functions of the transcription factor yin yang 1 in late embryonic development and cell cycle progression. Mol. Cell Biol..

[39] Wilkinson F.H., Park K., Atchison M.L. (2006). Polycomb recruitment to DNA in vivo by the YY1 REPO domain. Proc. Natl. Acad. Sci. USA.

[40] Satijn D.P.E., Hamer K.M., den Blaauwen J., Otte A.P. (2001). The Polycomb Group Protein EED Interacts with YY1, and Both Proteins Induce Neural Tissue in XenopusEmbryos. Mol. Cell. Biol..

[41] Atchison M.L. (2014). Function of YY1 in Long-Distance DNA Interactions. Front. Immunol.

[42] Weintraub A.S., Li C.H., Zamudio A.V., Sigova A.A., Hannett N.M., Day D.S., Abraham B.J., Cohen M.A., Nabet B., Buckley D.L. (2017). YY1 Is a Structural Regulator of Enhancer-Promoter Loops. Cell.

[43] Kumar N., Srivillibhuthur M., Joshi S., Walton K.D., Zhou A., Faller W.J., Perekatt A.O., Sansom O.J., Gumucio D.L., Xing J. (2016). A YY1-dependent increase in aerobic metabolism is indispensable for intestinal organogenesis. Development.

[44] Perekatt A.O., Valdez M.J., Davila M., Hoffman A., Bonder E.M., Gao N., Verzi M.P. (2014). YY1 is indispensable for Lgr5+ intestinal stem cell renewal. Proc. Natl. Acad. Sci. USA.

[45] Kleiman E., Jia H., Loguercio S., Su A.I., Feeney A.J. (2016). YY1 plays an essential role at all stages of B-cell differentiation. Proc. Natl. Acad. Sci. USA.

[46] Trabucco S.E., Gerstein R.M., Zhang H. (2016). YY1 Regulates the Germinal Center Reaction by Inhibiting Apoptosis. J. Immunol..

[47] Onder T.T., Kara N., Cherry A., Sinha A.U., Zhu N., Bernt K.M., Cahan P., Marcarci B.O., Unternaehrer J., Gupta P.B. (2012). Chromatin-modifying enzymes as modulators of reprogramming. Nature.

[48] Maestro R., Dei Tos A.P., Hamamori Y., Krasnokutsky S., Sartorelli V., Kedes L., Doglioni C., Beach D.H., Hannon G.J. (1999). Twist is a potential oncogene that inhibits apoptosis. Genes Dev..

[49] Seger Y.R., García-Cao M., Piccinin S., Cunsolo C.L., Doglioni C., Blasco M.A., Hannon G.J., Maestro R. (2002). Transformation of normal human cells in the absence of telomerase activation. Cancer Cell.

[50] Hannon G.J., Sun P., Carnero A., Xie L.Y., Maestro R., Conklin D.S., Beach D. (1999). MaRX: An approach to genetics in mammalian cells. Science.

[51] Ye J., Coulouris G., Zaretskaya I., Cutcutache I., Rozen S., Madden T.L. (2012). Primer-BLAST: A tool to design target-specific primers for polymerase chain reaction. BMC Bioinform..

[52] Davis C.A., Hitz B.C., Sloan C.A., Chan E.T., Davidson J.M., Gabdank I., Hilton J.A., Jain K., Baymuradov U.K., Narayanan A.K. (2018). The Encyclopedia of DNA elements (ENCODE): Data portal update. Nucleic Acids Res..

[53] Kent W.J., Sugnet C.W., Furey T.S., Roskin K.M., Pringle T.H., Zahler A.M., Haussler A.D. (2002). The Human Genome Browser at UCSC. Genome Res..

[54] Clough E., Barrett T. (2016). The Gene Expression Omnibus Database. Statistical Genomics.

[55] Ochsner S.A., Abraham D., Martin K., Ding W., McOwiti A., Kankanamge W., Wang Z., Andreano K., Hamilton R.A., Chen Y. (2019). The Signaling Pathways Project, an integrated ‘omics knowledgebase for mammalian cellular signaling pathways. Sci. Data.

[56] Koster J. R2: Genomics Analysis and Visualization Platform. http://r2.amc.nl.

[57] Lee H.-O., Hong Y., Etlioglu H.E., Cho Y.B., Pomella V., Van den Bosch B., Vanhecke J., Verbandt S., Hong H., Min J.-W. (2020). Lineage-dependent gene expression programs influence the immune landscape of colorectal cancer. Nat. Genet..

[58] EMBL-EBI Single Cell Expression Atlas. https://www.ebi.ac.uk/gxa/sc/home.

[59] KOBIC User-Friendly InteRface Tool to Explore Cell Atlas (URECA). http://ureca-singlecell.kr/.

