# Supplementary material for: YY1 Silencing Induces 5-Fluorouracil-Resistance and BCL2L15 Downregulation in Colorectal Cancer Cells: Diagnostic and Prognostic Relevance
Source: Int J Mol Sci. 2021 Aug 6;22(16):8481. doi: 10.3390/ijms22168481 (PMC8395225; doi:10.3390/ijms22168481)

Supplementary Materials

YY1 silencing induces 5-Fluorouracil-resistance and BCL2L15 downregulation in colorectal cancer cells: diagnostic and prognostic relevance

Silvia Vivarelli[1], Luca Falzone[2*], Saverio Candido[1,3], Benjamin Bonavida[4], and Massimo Libra[1,3*]

[1]Department of Biomedical and Biotechnological Sciences, University of Catania, Italy;

[2]Epidemiology Unit, IRCCS Istituto Nazionale Tumori "Fondazione G. Pascale", Naples, Italy;

[3]Research Center for Prevention, Diagnosis and Treatment of Cancer, University of Catania, Italy.

[4]Department of Microbiology, Immunology and Molecular Genetics, David Geffen School of Medicine, University of California, Los Angeles, California, USA.

[*]Co-corresponding authors

Table S1. Molecular and pathological features of the CRC cell line panel

***Feature*/Cell Line**	**HT29**	**SW620**	**HCT116**	**Caco2**
Organ	Colon	Colon	Colon ascendens	Colon
Disease	Colorectal adenocarcinoma	Colorectal adenocarcinoma	Colorectal carcinoma	Colorectal adenocarcinoma
Patient	44-Year-old female	51-Year-old male	48-Year-old male	48-Year-old male
Stage	Dukes' C	Dukes' C	Dukes' D	Dukes' B
Derived from	Primary tumor	Lymph node metastasis	Primary tumor	nd
MSI status	MSS	MSS	MSI	MSS
CIMP panel 1	+	+	+	+
CIMP panel 2	+	–	+	–
CIN	+	46	–	48
APC mut 1	p.E853* (substitution - nonsense)	p.Q1338* (substitution - nonsense)	p.?	p.Q1367* (substitution - nonsense)
APC mut 2	p.T1556Nfs*3 (insertion - frameshift)	p.?	p.?	p.?
CTNNB1 mut 1	wt	wt	p.S45del (deletion - in frame)	p.G245A
CTNNB1 mut 2	wt	wt	p.?	p.?
TP53 mut 1	p.R273H (substitution - missense) Gain of function	p.R273H (substitution - missense) Gain of function	wt	p.Glu204Ter (substitution - nonsense - KO)
TP53 mut 2	p.?	p.P309S	wt	p.Glu204Ter (substitution - nonsense - KO)
KRAS mut 1	wt	p.G12V (substitution - missense)	p.G13D (substitution - missense)	wt
KRAS mut 2	wt	p.?	p.?	wt
BRAF mut 1	p.Val600Glu (substitution - missense)	wt	p.Ser45del	wt
BRAF mut 2	p.?	wt	p.?	wt
PIK3CA mut 1	p.Pro449Thr (substitution - missense)	wt	p.His1047Arg	wt
PIK3CA mut 2	p.?	wt	p.?	wt
SMAD4 mut 1	p.Q311* (substitution - nonsense)	p.?	nd	p.D351H (substituion - missense)
SMAD4 mut 2	p.?	p.?	nd	p.?
CDKN2A mut 1	nd	nd	p.Arg24fs*20	nd
CDKN2A mut 2	nd	nd	p.Glu74fs*15	nd

Table S2. Whole Transcription Factors ChIP-seq binding-clusters within BCL2L15 promoter/enhancer (all experiments)

Experiment Name	Tissue	Binding Score	co-IP TF ID
**ATF2 IP - GM12878 cells**	lymphoid lineage, B lymphocytes, GM12878 cells	92	ATF2
**ATF3 IP**	epithelium, HCT116 cells	134	ATF3
**ATF3 IP | CAMPTO**	epithelium, HCT116 cells	311	ATF3
**BATF IP - GM12878 cells**	lymphoid lineage, B lymphocytes, GM12878 cells	182	BATF
**BCL11A IP - GM12878 cells**	lymphoid lineage, B lymphocytes, GM12878 cells	151	BCL11A
**BHLHE40 IP - GM12878 cells**	lymphoid lineage, B lymphocytes, GM12878 cells	117	BHLHE40
**CDX2 IP | 125DVD3**	epithelium, LS180 cells	94	CDX2
**CDX2 IP**	epithelium, LS180 cells	126	CDX2
**CEBPB IP - K562 cells**	myeloid lineage, myeloblasts, CML, K562 cells	141	CEBPB
**CEBPB IP**	multipotent, MSCs	166	CEBPB
**CEBPB IP**	myeloid lineage, macrophages, MV-4-11 cells	180	CEBPB
**CEBPB IP - HCT116 cells**	epithelium, HCT116 cells	186	CEBPB
**CEBPB IP - MCF7 cells**	epithelium, MCF-7 cells	209	CEBPB
**CEBPB ChIP-Seq - K562**	myeloid lineage, myeloblasts, CML, K562 cells	361	CEBPB
**CEBPB IP**	multipotent, MSCs	419	CEBPB
**CEBPB IP**	multipotent, MSCs	486	CEBPB
**E2F3 IP**	epithelium, LoVo cells	155	E2F3
**EBF1 IP - GM12878 cells**	lymphoid lineage, B lymphocytes, GM12878 cells	103	EBF1
**ELF3 IP | KLF5 KO**	duct, epithelium, CFPAC-1 cells	273	ELF3
**ELF3 IP**	duct, epithelium, CFPAC-1 cells	586	ELF3
**ELF3 IP**	duct, epithelium, CFPAC-1 cells	731	ELF3
**EOMES IP - Rep 3**	pluripotent, ESCs, HUES64 cells	266	EOMES
**ETS1 IP**	lymphoid lineage, lymphoblasts, ALL, CUTLL1 cells	183	ETS1
**ETV5 IP**	epithelium, LoVo cells	146	ETV5
**FOSL1 IP - HCT116 cells**	epithelium, HCT116 cells	524	FOSL1
**FOXA1 ChIP-Seq - Rep 1**	epithelium, ZR-75-1 cells	105	FOXA1
**DHT | FOXA1 ChIP-Seq**	epithelium, LNCaP cells	110	FOXA1
**FOXA1 ChIP-Seq**	epithelium, LNCaP cells	111	FOXA1
**FOXA1 IP - ES D7**	pluripotent, ESCs, Cyt49 cells	136	FOXA1
**FOXA1 ChIP-Seq**	epithelium, LNCaP-abl cells	156	FOXA1
**FOXA1 IP - Rep 1**	pluripotent, ESCs, HUES64 cells	164	FOXA1
**CREB1 siRNA | FOXA1 ChIP-Seq - Rep 1**	epithelium, LNCaP-abl cells	193	FOXA1
**FOXA1 ChIP-Seq - LNCaP-abl**	epithelium, LNCaP-abl cells	194	FOXA1
**FOXA1 IP - T47D cells**	epithelium, T47D cells	201	FOXA1
**DEX | FOXA1 ChIP-Seq - Rep 2**	epithelium, ZR-75-1 cells	221	FOXA1
**FOXA1 ChIP-Seq - Rep 2**	epithelium, LNCaP-abl cells	224	FOXA1
**CREB1 siRNA | FOXA1 ChIP-Seq - Rep 2**	epithelium, LNCaP-abl cells	244	FOXA1
**FOXA1 IP**	duct, epithelium, CFPAC-1 cells	295	FOXA1
**FOXA1 IP**	duct, epithelium, CFPAC-1 cells	308	FOXA1
**FOXA1 ChIP-Seq**	epithelium, LNCaP cells	359	FOXA1

**FOXA1 IP - Rep 5**	pluripotent, ESCs, HUES64 cells	540	FOXA1
**FOXA2 IP - Rep 5**	pluripotent, ESCs, HUES64 cells	193	FOXA2
**FOXA2 IP - Rep 1**	pluripotent, ESCs, HUES64 cells	289	FOXA2
**FOXA2 IP - Rep 1**	endodermal cells	398	FOXA2
**FOXM1 IP - ECC1 cells**	endometrium, epithelium, ECC-1 cells	139	FOXM1
**FOXP1 IP**	epithelium, LoVo cells	115	FOXP1
**GABPA IP - Jurkat, Rep 1**	lymphoid lineage, T lymphocytes, Jurkat	161	GABPA
**FOXA1 KD | GATA2 ChIP-Seq**	epithelium, LNCaP cells	179	GATA2
**17BE2 | GATA3 ChIP-Seq**	epithelium, MCF-7 cells	90	GATA3
**GATA3 IP - T47D cells**	epithelium, T47D cells	158	GATA3
**GATA3 IP - SK-N-SH cells**	other, neuroblastoma cells, SK-N-SH cells	198	GATA3
**GATA4 IP - Rep 11**	pluripotent, ESCs, HUES64 cells	118	GATA4
**GATA4 IP - Rep 8**	pluripotent, ESCs, HUES64 cells	151	GATA4
**GATA4 IP - Rep 10**	pluripotent, ESCs, HUES64 cells	164	GATA4
**GATA4 IP - AGS**	epithelium, AGS cells	186	GATA4
**GATA4 IP - Rep 9**	pluripotent, ESCs, HUES64 cells	187	GATA4
**HNF1B IP**	duct, epithelium, CFPAC-1 cells	416	HNF1B
**HOXA4 IP**	epithelium, LoVo cells	133	HOXA4
**IRF1 IP**	duct, epithelium, CFPAC-1 cells	180	IRF1
**IRF1 IP**	epithelium, LoVo cells	323	IRF1
**IRF1 IP - Rep 3**	lymphoid lineage, T lymphocytes, CD4+	373	IRF1
**JUN IP**	epithelium, LoVo cells	134	JUN
**JUN IP - Rep 2**	epithelium, MDA-MB-231 cells	184	JUN
**JUN IP - Rep 1**	epithelium, MDA-MB-231 cells	329	JUN
**JUND IP**	smooth muscle, whole	189	JUND
**JUND IP - HCT116 cells**	epithelium, HCT116 cells	766	JUND
**KLF5 IP - KATOIII**	epithelium, KATO III cells	125	KLF5
**KLF5 IP**	duct, epithelium, CFPAC-1 cells	383	KLF5
**MAX IP - HCT116 cells**	epithelium, HCT116 cells	172	MAX
**MYB IP - Jurkat, Rep 3**	lymphoid lineage, T lymphocytes, Jurkat	135	MYB
**MYB IP - Jurkat, Rep 1**	lymphoid lineage, T lymphocytes, Jurkat	194	MYB
**MYB IP - Jurkat, Rep 5**	lymphoid lineage, T lymphocytes, Jurkat	217	MYB
**MYB IP - Jurkat, Rep 4**	lymphoid lineage, T lymphocytes, Jurkat	529	MYB
**NFYB IP - GM12878 cells**	lymphoid lineage, B lymphocytes, GM12878 cells	214	NFYB
**OTX2 IP - Rep 8**	pluripotent, ESCs, HUES64 cells	165	OTX2
**OTX2 IP - Rep 6**	pluripotent, ESCs, HUES64 cells	197	OTX2
**SP1 IP - GM12878 cells**	lymphoid lineage, B lymphocytes, GM12878 cells	79	SP1
**SP1 IP - HCT116 cells**	epithelium, HCT116 cells	545	SP1
**SPI1 IP - HL-60 cells, Rep 4**	myeloid lineage, promyelocytes, APL, HL-60 cells	109	SPI1
**SPI1 IP - U-937 cells, Rep 1**	myeloid lineage, monocytes, U-937 cells	180	SPI1
**SPI1 IP | RUNX1 KD**	myeloid lineage, myeloblasts, AML, kasumi-1 cells	204	SPI1
**SPI1 IP - U-937 cells, Rep 4**	myeloid lineage, monocytes, U-937 cells	235	SPI1
**SPI1 IP - GM12891 cells**	lymphoid lineage, B lymphocytes, GM12891 cells	259	SPI1
**SPI1 IP**	myeloid lineage, macrophages, unspecified	264	SPI1
**SPI1 IP**	myeloid lineage, myeloblasts, AML, kasumi-1 cells	310	SPI1

**SPI1 IP**	myeloid lineage, macrophages, unspecified	339	SPI1
**SPI1 IP - GM12878 cells**	lymphoid lineage, B lymphocytes, GM12878 cells	342	SPI1
**SPI1 IP - HL cells**	myeloid lineage, promyelocytes, APL, HL-60 cells	379	SPI1
**SPI1 IP**	myeloid lineage, macrophages, unspecified	643	SPI1
**SPI1 IP**	myeloid lineage, macrophages, unspecified	849	SPI1
**SPI1 IP**	myeloid lineage, macrophages, unspecified	1046	SPI1
**SPI1 IP**	myeloid lineage, macrophages, unspecified	1133	SPI1
**SPI1 IP**	myeloid lineage, macrophages, unspecified	1197	SPI1
**SRF IP - HCT116 cells**	epithelium, HCT116 cells	99	SRF
**TAL1 IP**	epithelium, LoVo cells	127	TAL1
**TCF12 IP - ECC1 cells**	endometrium, epithelium, ECC-1 cells	142	TCF12
**TCF12 IP - SK-N-SH cells**	other, neuroblastoma cells, SK-N-SH cells	207	TCF12
**TEAD4 IP - HCT116 cells**	epithelium, HCT116 cells	279	TEAD4
**TEAD4 IP - ECC1 cells**	endometrium, epithelium, ECC-1 cells	390	TEAD4
**ZNF384 IP - GM12878 cells**	lymphoid lineage, B lymphocytes, GM12878 cells	81	ZNF384

Table S3. GEO DataSets features, normality and correlation analyses

GEO Dataset Name	GSE ID	Year	Patients	YY1 Normality test	BCL2L15 Normality test	Pearson R	*Pearson P*	Spearman R	*Spearman P*
**EXPO**	GSE2109	2005	315	0.4169 (Y)	<0.0001 (N)	0.0439	*0.4368*	0.0408	*0.4710*
**Watanabe**	GSE4554	2006	84	<0.0001 (N)	<0.0001 (N)	0.1820	*0.0976*	0.1949	*0.0756*
**Marra**	GSE8671	2007	32	0.6751 (Y)	0.1307 (Y)	-0.1260	*0.4847*	-0.0955	*0.6029*
**Jorissen**	GSE13294	2009	155	0.0747 (Y)	<0.0001 (N)	0.0052	*0.9490*	0.0211	*0.7936*
**Smith**	GSE17538	2009	232	<0.0001 (N)	0.3461 (Y)	0.0577	*0.3815*	0.0723	*0.2722*
**Sieber**	GSE14333	2010	289	0.0025 (N)	<0.0001 (N)	0.0082	*0.8890*	0.0206	*0.7270*
**Sugihara**	GSE21510	2011	148	0.8457 (Y)	<0.0001 (N)	-0.0104	*0.8997*	0.0157	*0.8497*
**Yagi**	GSE28702	2011	83	0.7817 (Y)	0.339 (Y)	0.0096	*0.9312*	0.0905	*0.4159*
**Olschwang**	GSE37892	2012	130	0.0122 (N)	0.1917 (Y)	0.1169	*0.1853*	0.1923	*0.0284*
**Wessels**	GSE35896	2013	62	0.2448 (Y)	<0.0001 (N)	-0.1213	*0.3475*	-0.0368	*0.7766*
**Clary**	GSE41568	2016	133	0.6228 (Y)	0.0052 (N)	0.1024	*0.2409*	0.1232	*0.1578*
**Sieber**	GSE75316	2016	58	0.6715 (Y)	<0.0001 (N)	0.0641	*0.6329*	-0.0305	*0.8200*

Table S4. Primers used in the RT-PCR experiments

GENE ID	Primer Forward	Primer Reverse	bp
BCL2	TGAACTGGGGGAGGATTGTG	CGTACAGTTCCACAAAGGCA	183
BCL2L1 XS	AGCTTTGAACAGGATACTTTTGTGG	GGTGGGAGGGTAGAGTGGAT	183
BCL2L1 XL	CTGTGCGTGGAAAGCGTAGA	GCTGCTGCATTGTTCCCATAG	155
BCL2L2	CACCCAGGTCTCCGATGAAC	GCTGTGAACTCCGCCCAG	210
BCL2L3	TTTTCAGCGACGGCGTAACA	CAAACCCATCCCAGCCTCTTT	189
BCL2L4	CCCCGAGAGGTCTTTTTCCG	TGGTTCTGATCAGTTCCGGC	145
BCL2L7	GATCCCGGCAGGCTGATCC	GTAGCTGCGGAAAACCTCCT	156
BCL2L8	CTTTAAGAAGGGACTTCCTCGC	GTGGAGTTTCGGGATGTGGA	163
BCL2L15	ACCTGGTGTGCTCAGGATTC	TCCAGATTTTCCCAACCTCCC	194
BIRC3	TCTGGGCAGCAGGTTTACAA	GCATTCTTTGGATAGTAAAACACCA	191
BIRC4	TGTCCTGGCGCGAAAAGGT	CGTGCCAGTGTTGATGCTGA	190
BIRC5	CAAGGACCACCGCATCTCTA	TGTTCCTCTATGGGGTCGTCA	189
BIK	CCGCCAGAGGAGAAATGTCTGA	TCCTCCATAGGGTCCAGGTC	145
TP53	CCCCTCCTCAGCATCTTATCC	GTACAGTCAGAGCCAACCTCAG	124
YY1	GAGAGAACTCACCTCCTGAT	GGCTTCTCTCCAGTATGAAC	325
GAPDH	AGAAGGCTGGGGCTCATTTG	AGGGGCCATCCACAGTCTTC	258

Table S5. Antibodies used for immunoblot analyses

Antibody	Dilution in blocking buffer	Brand	ID
**anti-YY1**	1:1 000 dilution	Cell Signaling Technology, Danvers, MA, USA	CST-2185
**anti-Bfk**	1:1 000 dilution	Abcam, Cambridge, UK	Ab-18493
**anti-β-Actin**	1:10 000 dilution	Sigma-Aldrich, St. Louis, MO, USA	A1978
**anti-GAPDH**	1:1 000 dilution	Santa Cruz Biotech,Dallas, TX, USA	sc-137179
**anti-cleaved-Caspase-3**	1:1 000 dilution	Cell Signaling Technology, Danvers, MA, USA	CST-9664
**anti-Flag-M2**	1:1 000 dilution	Sigma-Aldrich, St. Louis, MO, USA	F1804
**Goat Anti-Rabbit IgG Antibody, Fc, HRP conjugate**	1:3 000 dilution	Chemicon International, Fisher Scientific, Waltham, MA, USA	AP156P
**Goat Anti-Mouse IgG Antibody, Fc, HRP conjugate**	1:3 000 dilution	Chemicon International, Fisher Scientific, Waltham, MA, USA	AP127P

Figure S1. Sq-RT-PCR densitometry analysis of YY1 putative transcriptional targets in the apoptotic pathway in CRC cells panel (HCT-116, Caco-2, HT-29 SW620). Signals are normalized with GAPDH, used as housekeeping. Values are presented as Mean ± SD.

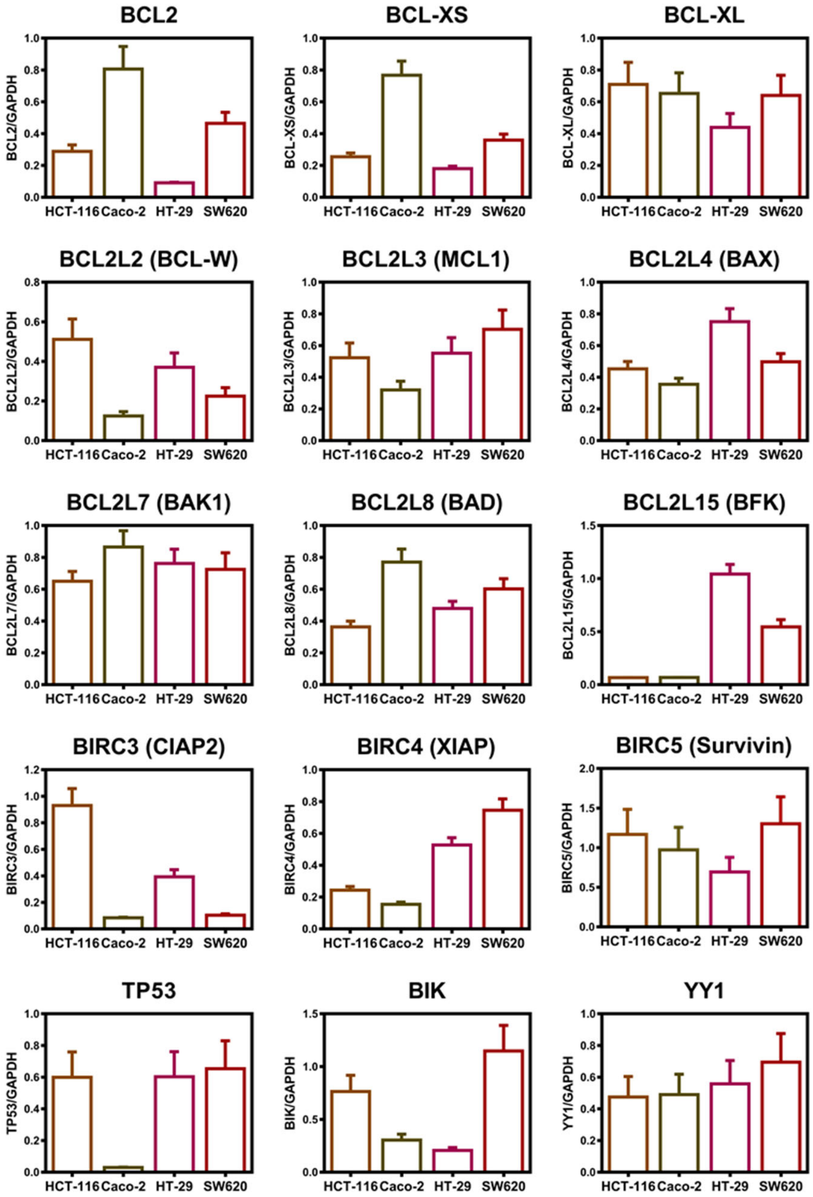

Figure S2. Sq-RT-PCR densitometry analysis of YY1 putative transcriptional targets in the apoptotic pathway in HT-29 cells (CTRL, KD-01, KD-02). Signals are normalized with GAPDH, used as housekeeping. Values are presented as Mean ± SD. **P<0.05; **P<0.01; ***P<0.001; ****P<0.0001*.

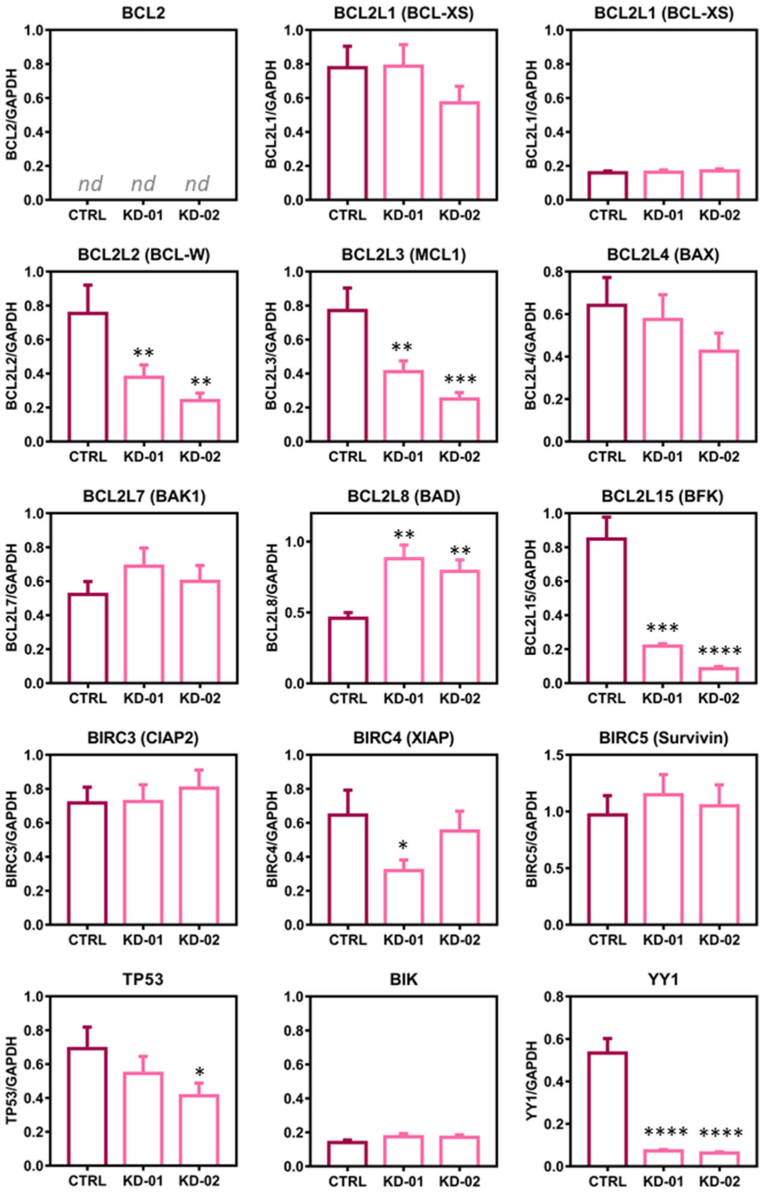

Figure S3. Sq-RT-PCR densitometry analysis of YY1 putative transcriptional targets in the apoptotic pathway in SW620 cells (CTRL, KD-01, KD-02). Signals are normalized with GAPDH, used as housekeeping. Values are presented as Mean ± SD. **P<0.05; **P<0.01; ***P<0.001.*

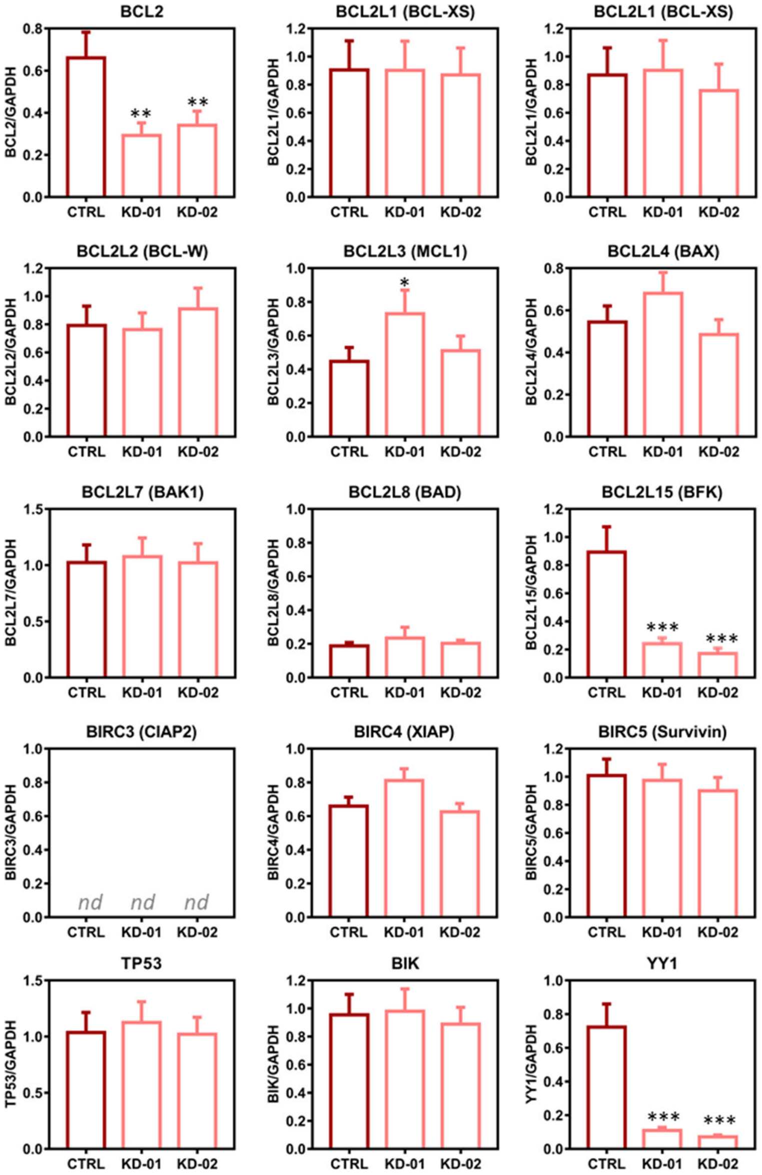

Figure S4. Whole Transcription Factors ChIP-seq binding-clusters within BCL2L15 regulatory region (±10Kb from Transcription Starting Site, TSS). Binding score arbitrary units (from 0 to 1200). Highlighted blue: Transcription factor class, Black: Transcription factor subclass, Light grey: Transcription factor gene name. Each red dot is an independent ChIP-seq experiment and indicates the intersection between the specific transcription factor gene name and its relative binding score to the BCL2L15 regulatory region.

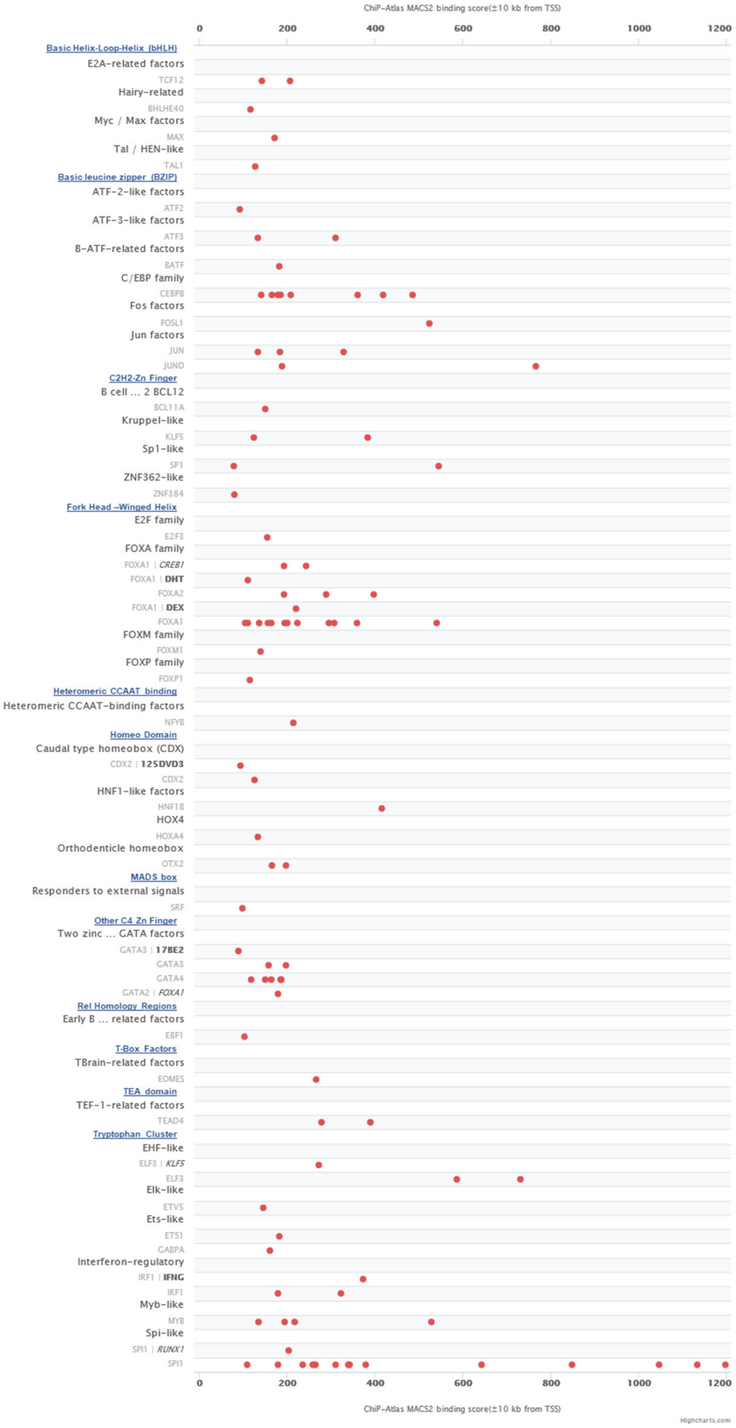

Figure S5. Whole Transcription Factors ChIP-seq binding-clusters, in gastro-intestinal cancer cells, within BCL2L15 regulatory region (±10Kb from Transcription Starting Site, TSS). Binding score arbitrary units (from 0 to 1200). Highlighted blue: Transcription factor class, Black: Transcription factor subclass, Light grey: Transcription factor gene name. Each red dot is an independent ChIP-seq experiment and indicates the intersection between the specific transcription factor gene name and its relative binding score to the BCL2L15 regulatory region.

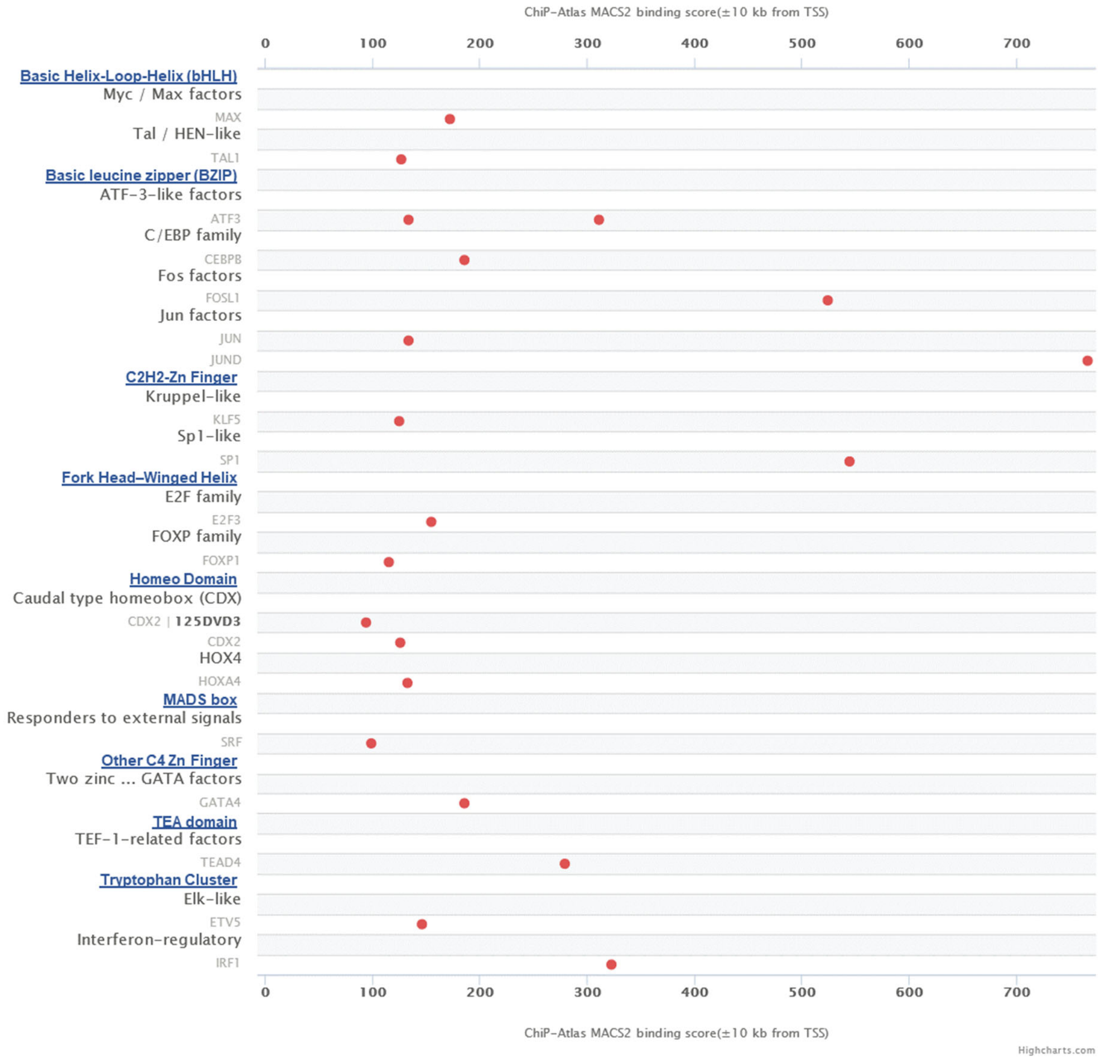

Figure S6. Correlation plots between YY1 and BCL2L15 expression in twelve CRC GEO datasets. YY1 probe: YY1 200047_s_at; BCL2L15 probe: BCL2L15 236979_at.

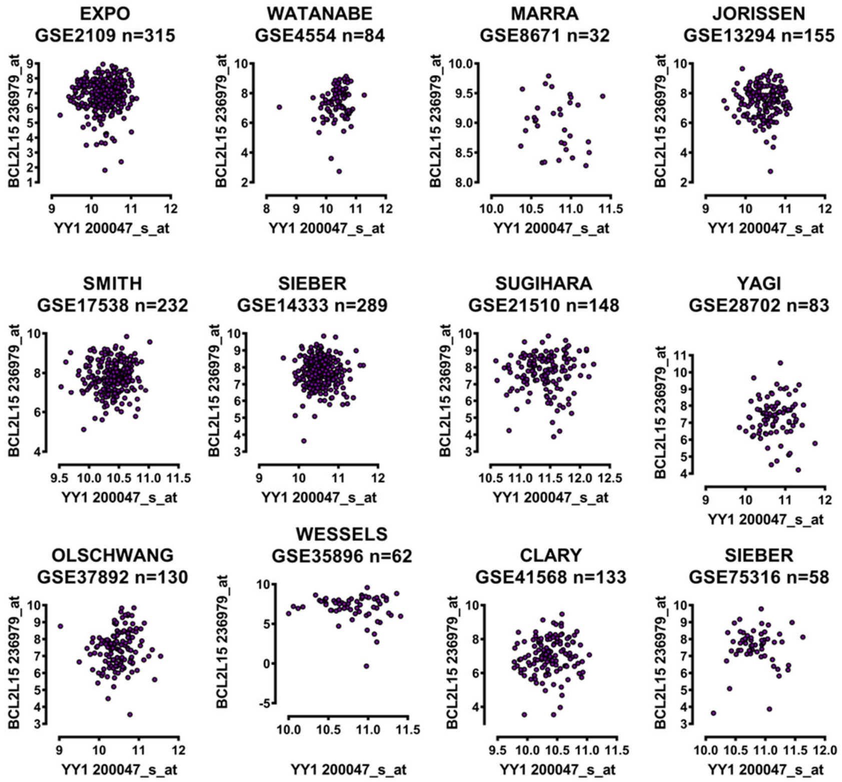

Figure S7. Correlation between YY1 expression and stratification of samples according to genetic mutational status in GSE35896. Dot plots with median of YY1 2log expression in CRC samples of: **A.** APC gene (WT vs. MUT). **B.** BRAF gene (WT vs. MUT). **C.** KRAS gene (WT vs. MUT). **D.** PIK3CA gene (WT vs. MUT). **E.** PTEN gene (WT vs. MUT). **F.** TP53 gene (WT vs. MUT). n.s.= not significant.

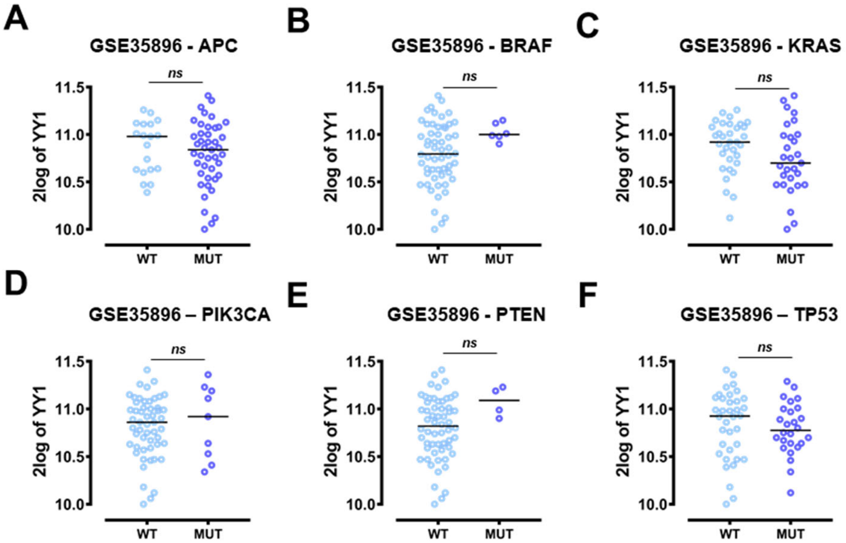

Figure S8. T-SNE plots of non-tumoral cells: YY1 and BCL2L15 expression. **A.** t-SNE plot of stromal cells (clusters). **B.** t-SNE plot of stromal cells (YY1 expression). **C.** t-SNE plot of stromal cells (BCL2L15 expression). **D.** t-SNE plot of myeloid cells (clusters). **E.** t-SNE plot of myeloid cells (YY1 expression). **F.** t-SNE plot of myeloid cells (BCL2L15 expression). **G.** t-SNE plot of T cells (clusters). **H.** t-SNE plot of T cells (YY1 expression). **I.** t-SNE plot of T cells (BCL2L15 expression). **J.** t-SNE plot of B cells (clusters). **K.** t-SNE plot of B cells (YY1 expression). **L.** t-SNE plot of B cells (BCL2L15 expression).

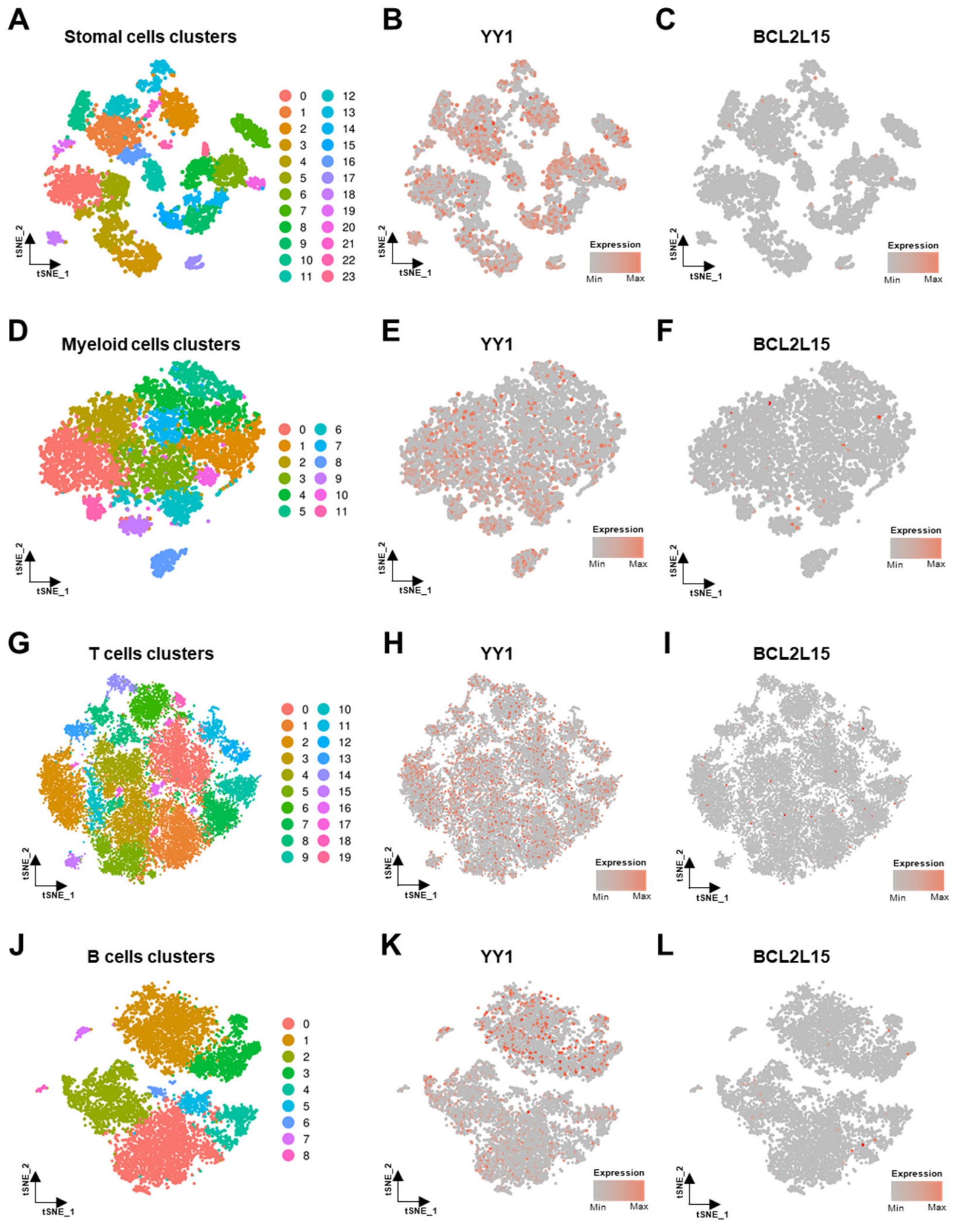

Figure S9. YY1 antibody mono-specificity. The YY1-mAb used for WB analyses selectively recognizes YY1 and not the homologous YY2. Flag-tagged human recombinant YY2 (hr-YY2) purified protein was loaded as control. Anti-FLAG antibody was used to detect hr-YY2.

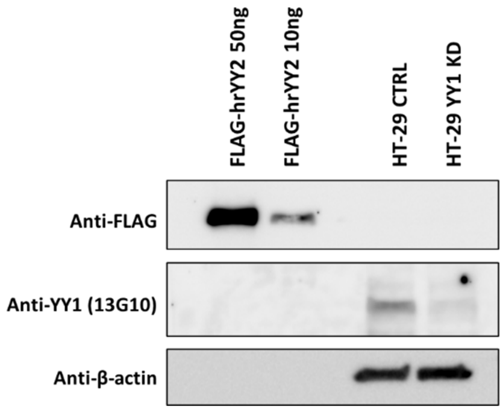

Supplement: Supplementary file 1 [file ijms-22-08481-s001.zip › ijms-1307663-supplementary.pdf]
